# Coordination‐Tuned Iridium Single‐Atom Nanozymes Boost Multienzyme Activity for Colorimetric Sensing

**DOI:** 10.1002/advs.76542

**Published:** 2026-07-29

**Authors:** Tao Li, Xinyu Zhang, Jiashan Xia, Mengyu Wu, Cong Liu, Yapei Sun, Wanjiang Zhao, Min Qian, Wei Wang, Weixia Duan, Shangcheng Xu

**Affiliations:** ^1^ Chongqing Key Laboratory of Prevention and Treatment for Occupational Diseases and Poisoning Chongqing Municipal Health Commission Key Laboratory for Emergency Poisoning Detection and Acute Care The First Affiliated Hospital of Chongqing Medical and Pharmaceutical College Chongqing China; ^2^ Center For Global Health School of Public Health Nanjing Medical University Nanjing China; ^3^ Department of Occupational and Environmental Health School of Public Health Chongqing Medical University Chongqing China

**Keywords:** asymmetric coordination engineering, colorimetric sensing, multienzyme‐like activity, single‐atom nanozymes

## Abstract

Precisely manipulating the coordination environment in single‐atom nanozymes (SAzymes) remains a critical challenge in breaking the intrinsic activity ceiling, thus limiting the multifunctionality of current enzyme‐mimetic catalysts. In this study, iridium SAzymes featuring an asymmetric Ir–N_3_S_1_ coordination motif (Ir–S/N–C) were prepared by adding sulfur to a traditional Ir–N–C framework. Density functional theory calculations revealed that this symmetry‐breaking coordination design enabled the fine modulation of the local electronic structure of the isolated iridium centers, which upshifted the *d*‐band center and substantially reduced the energy barrier for O_2_ activation. Ir–S/N–C exhibited markedly enhanced oxidase‐ and peroxidase‐like activities, and a glutathione‐oxidase‐like functionality emerged, thus achieving integrated multienzyme‐like catalysis within a single‐atom platform. Notably, the strong intrinsic oxidase activity allowed the construction of a self‐sufficient H_2_O_2_‐free colorimetric sensing system for antioxidants and organophosphate pesticides, delivering high sensitivity and selectivity in complex sample matrices. Overall, this study demonstrated asymmetric sulfur coordination as a promising coordination‐engineering strategy for modulating the electronic structure of single‐atom sites and highlighted its effectiveness in developing high‐performance multifunctional SAzymes.

## Graphical abstract

Breaking the coordination symmetry of Ir single‐atom sites through asymmetric Ir–N_3_S_1_ engineering tailors interfacial electron transfer, oxygen activation, and multienzyme‐like catalytic pathways. Operando spectroscopy and theoretical modeling uncover the atomic‐level origin of activity enhancement, providing a strategy for designing multifunctional nanozymes for practical biosensing and environmental analysis.

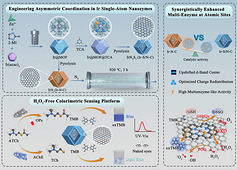

## 1 Introduction

The accurate and sensitive detection of biomolecules and environmental contaminants is critical for disease diagnosis, food safety, and monitoring. Among various biosensing systems, colorimetric methods based on artificial nanozymes have attracted considerable attention owing to their simplicity, visual readout, and potential for field deployment [1, 2, 3, 4]. Despite this progress, many classical nanozymes suffer from limited catalytic activity, insufficient substrate selectivity, and modest signal amplification, restricting their broad practical use.

Single‐atom nanozymes (SAzymes), which feature atomically dispersed metal centers anchored on supports such as carbon matrices or metal–organic frameworks, have emerged as effective platforms for artificial enzymatic catalysis [5, 6]. Compared with conventional nanozymes, SAzymes offer higher metal utilization, more precisely defined active sites, and greater freedom to tune the local coordination environment, which is crucial for regulating catalytic activity and selectivity [7, 8]. Recent studies have shown that SAzymes can be engineered to exhibit diverse enzyme‐mimicking functions, including peroxidase (POD)‐ [9], oxidase (OXD)‐ [10], catalase‐ [11], phosphatase‐ [12], and superoxide‐dismutase‐like activities [13], highlighting their versatility in biosensing and catalytic applications. Tuning the local coordination environment of the single‐atom centers is critical for modifying the activity and selectivity of SAzymes [14]. For instance, heteroatom doping (e.g., B, S, Cl, and P) or adjusting the coordination number (e.g., Mn–N_3_ vs Mn–N_4_) has been shown to significantly enhance the catalytic efficiency and multienzyme functionality, enabling more sensitive and selective colorimetric biosensing [15, 16, 17, 18, 19, 20, 21, 22]. Coordination modulation not only optimizes the charge redistribution and electronic coupling between the metal center and surrounding ligands but also fine‐tunes the adsorption and activation of catalytic intermediates, improving both efficiency and selectivity [23, 24, 25]. These advancements prove that coordination engineering can effectively create high‐performance SAzymes with customizable electronic structures and catalytic functions.

Ir is distinguished by its variable valence states (Ir^0^, Ir^3+^, and Ir^4+^), strong redox properties, and outstanding chemical stability, making it a promising candidate for SAzymes [26, 27]. Ir–N–C SAzymes, particularly Ir–N_4_, have large *d*‐band centers and partially filled *d*‐orbitals, which lower their activation barriers for substrates such as H_2_O_2_ [28]. To maintain dual‐enzyme reaction loops, Ir–N–C SAzymes are combined with glucose OXD to create self‐amplified cascades in which the Ir sites concurrently display POD and catalase activities [27]. Ir‐based SAzymes have also shown great potential in biosensing applications. Wang et al. [29] loaded a high density of Ir(III) sites onto graphene oxide (GO) to construct an Ir(III)/GO SAzyme with high POD‐like activity, enabling the colorimetric detection of organophosphorus pesticides (OPs). Our group [30] synthesized single‑atom Ir‑doped carbon dots via in situ pyrolysis, achieving high POD‑like activity (≈178.8 U mg^−1^) for detecting mercury ions. However, Ir‐based SAzymes with precisely controlled coordination structures and multienzyme‐like activity have rarely been reported, highlighting the urgent need for strategies that can tailor their coordination environments for versatile catalytic applications.

**SCHEME 1:**
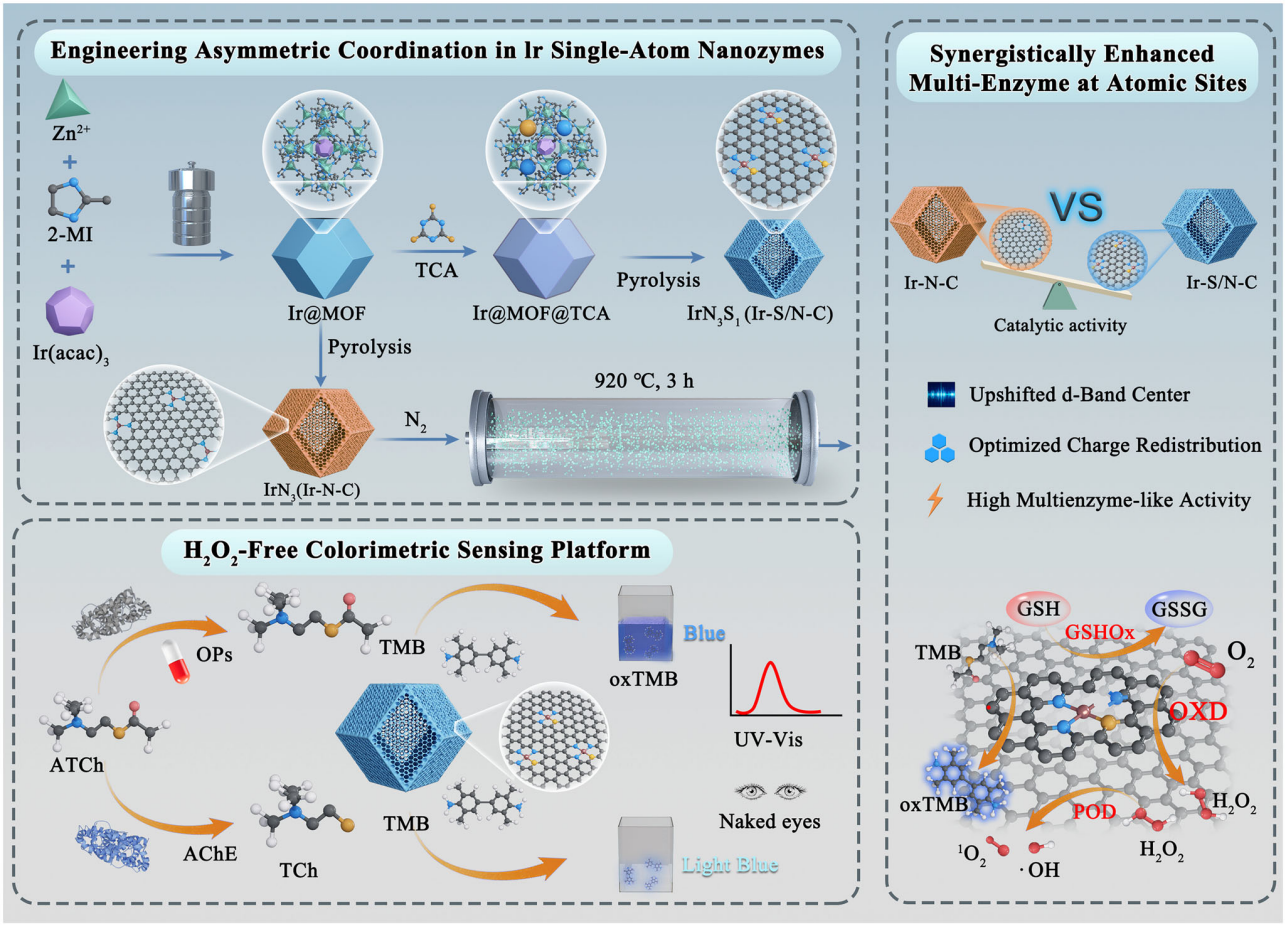
Fabrication process of the Ir–S/N–C SAzymes and their subsequent application in amplified colorimetric sensing.

In this study, a rational coordination‐engineering technique was used to synthesize Ir‐based SAzymes with asymmetric Ir–N_3_S active sites (denoted as Ir–S/N–C). By incorporating S atoms into the Ir–N–C framework, the local coordination environment of Ir was precisely modulated, as confirmed by aberration‐corrected high‐angle annular dark‐field–scanning transmission electron microscopy (HAADF–STEM) and x‐ray absorption near‐edge structure (XANES) analysis. This design achieved three key innovations: (i) a well‐defined Ir–N_3_S configuration disrupted the symmetry of conventional Ir–N_3_ sites; (ii) multienzyme activities (OXD, POD, and glutathione (GSH) oxidase (GSHOx)) were integrated within a single atomic architecture; and (iii) a self‐sufficient, H_2_O_2_‐free colorimetric sensing platform for antioxidants and OPs was developed. Density functional theory (DFT) calculations revealed that S coordination induced electron redistribution and decreased the Bader charge on Ir, optimizing the *d*‐band center and promoting O_2_ activation. These features endowed the Ir–S/N–C SAzyme with excellent catalytic efficiency, remarkable stability, and broad functional adaptability. Furthermore, the inherent OXD‐like activity allowed for the highly sensitive detection of both antioxidant substances (ascorbic acid (AA), cysteine (Cys), and GSH and acetylcholinesterase (AChE)‐targeting OPs without the need for external oxidants such as H_2_O_2_ (Scheme 1). Taken together, these findings demonstrated that asymmetric N/S coordination in Ir–S/N–C SAzymes could effectively modulate the electronic structure of Ir single‐atom sites within this system, offering a feasible coordination‐engineering approach for enhancing multienzyme‐like activity and facilitating self‐sufficient colorimetric biosensing.

## 2 Results and Discussion

### 2.1 Fabrication and Morphology of Ir–S/N–C SAzymes

**FIGURE 1:**
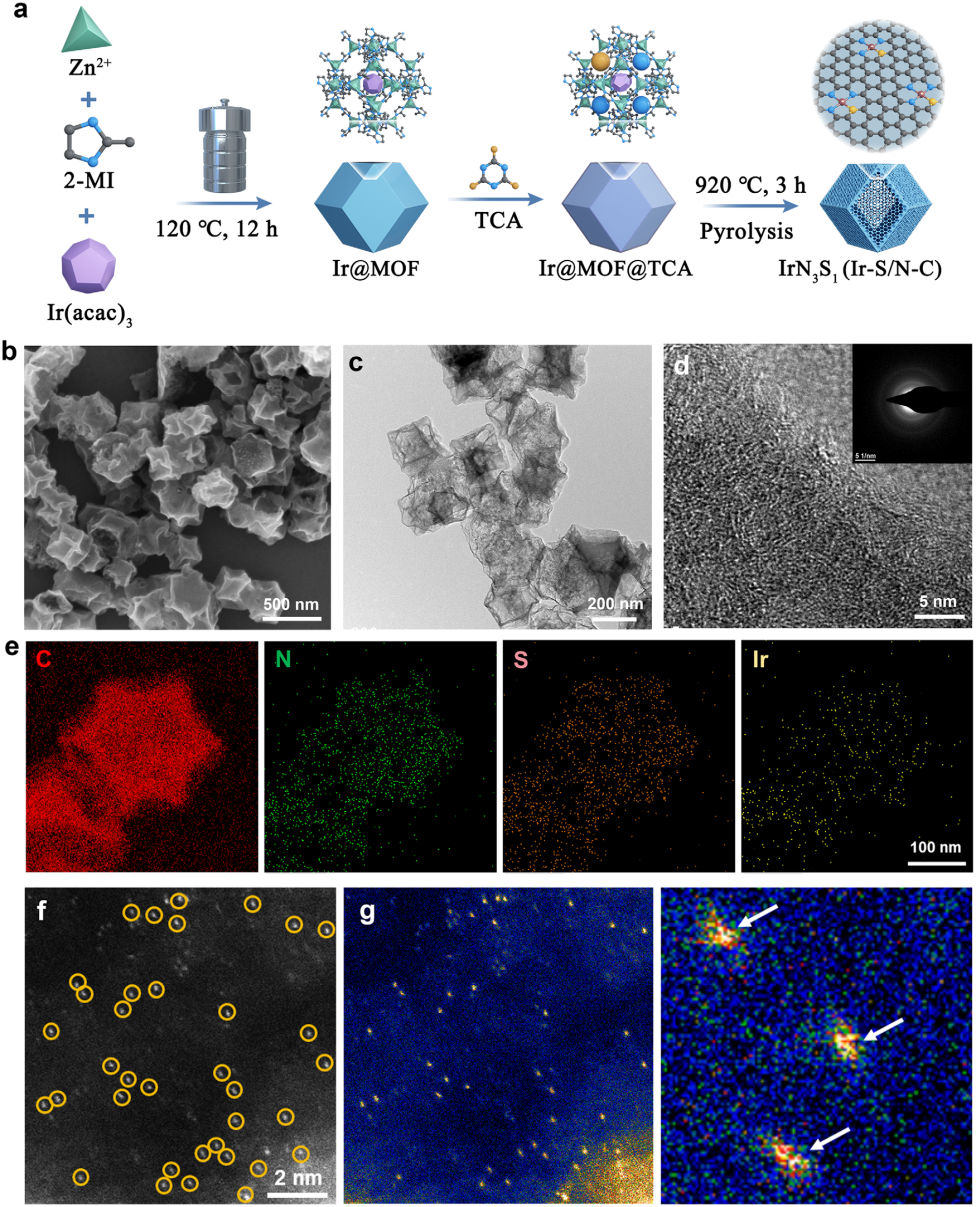
Synthesis and structural characterization of the Ir–S/N–C SAzymes. (a) Schematic of the synthesis strategy of the Ir–S/N–C SAzymes; 2‐methylimidazole: 2‐MI. (b) SEM, (c) TEM, and (d) HRTEM images (inset: SAED pattern) of the Ir–S/N–C SAzymes. (e) Corresponding EDS mapping of the Ir–S/N–C SAzymes. C, red; N, green; S, pink; Ir, yellow. (f) Atomic‐level HAADF–STEM image of the Ir–S/N–C SAzymes; yellow circles indicate Ir atoms. (g) Corresponding surface intensity map and enlarged surface intensity map of (g); the yellow dots are Ir atoms.

A typical synthetic route for the Ir–S/N–C SAzymes is illustrated in Figure 1a. First, Ir(acac)_3_ was encapsulated in ZIF‐8 via a host–guest strategy at 120°C in an autoclave, yielding a uniform Ir‐doped ZIF‐8 precursor (Ir‐ZIF‐8) [31]. As shown in Figure S1 and S2, Ir‐ZIF‐8 retained a homogeneous rhombic dodecahedral morphology, with an average diameter of 250 nm. Energy‐dispersive x‐ray spectroscopy (EDS) elemental mapping confirmed the uniform distribution of Zn, C, N, and Ir throughout the framework. The powder x‐ray diffraction (XRD) patterns matched the characteristic peaks of pristine ZIF‐8, indicating that Ir incorporation did not disrupt the host crystalline structure (Figure S3) [32]. Subsequently, a core–shell‐structured composite, Ir‐ZIF‐8@thioglycolic acid (TCA), was fabricated by coating Ir‐ZIF‐8 nanoparticles with TCA, exploiting the strong coordination interactions between thiol groups and both Zn^2+^ and Ir^3+^ ions. Transmission electron microscopy (TEM) images show that the composite retained its polyhedral morphology (∼250 nm) after coating although its surface became noticeably rougher (Figure S4a). EDS mapping further confirmed that S was successfully incorporated into the framework (Figure S4b). Finally, pyrolysis at 920°C for 3 h in a N_2_ atmosphere resulted in a S–N codoped carbon support with atomically dispersed Ir single atoms, denoted Ir–S/N–C (Figure 1a). For comparison, three control materials were prepared under the same conditions: N‐doped carbon without Ir (N–C), S/N‐codoped carbon without Ir (S/N–C), and Ir–N–C without S doping.

The morphologies and structures of the synthesized Ir–S/N–C SAzymes were thoroughly investigated using various techniques. Scanning electron microscopy (SEM) and TEM images revealed that the materials largely retained their original dodecahedral geometry although the surface became noticeably rough and porous after high‐temperature pyrolysis (Figure 1b,c). High‐resolution TEM (HRTEM) and selected‐area electron diffraction (SAED) patterns confirmed the amorphous nature of the carbon matrix and the absence of crystalline Ir nanoparticles (Figure 1d). Additionally, EDS elemental mapping indicated that C, N, S, and Ir were homogeneously distributed throughout the nanostructure (Figure 1e). TEM and SEM demonstrated similar dodecahedral shapes and porous surfaces for the control samples (N–C, S/N–C, and Ir–N–C; Figures S5–S7). Notably, aberration‐corrected atomic‐resolution HAADF–STEM images showed that isolated Ir atoms were atomically dispersed (highlighted by yellow circles, Figure 1f). Intensity mapping further validated the uniform spatial distribution of single Ir atoms within the S/N‐codoped carbon matrix (Figure 1g). A comparable Ir single‐atom distribution was observed for the traditional Ir–N–C SAzyme (Figure S8). Using inductively coupled plasma–optical emission spectrometry (ICP–OES), the Ir content of Ir–S/N–C was calculated to be 0.17 wt.%. Taken together, these findings verified that the Ir–S/N–C SAzymes containing single Ir atoms were successfully constructed; further research is required to understand their local coordination environment, electronic structure, and active‐site configuration.

### 2.2 Fine Structure of Ir–S/N–C SAzymes

**FIGURE 2:**
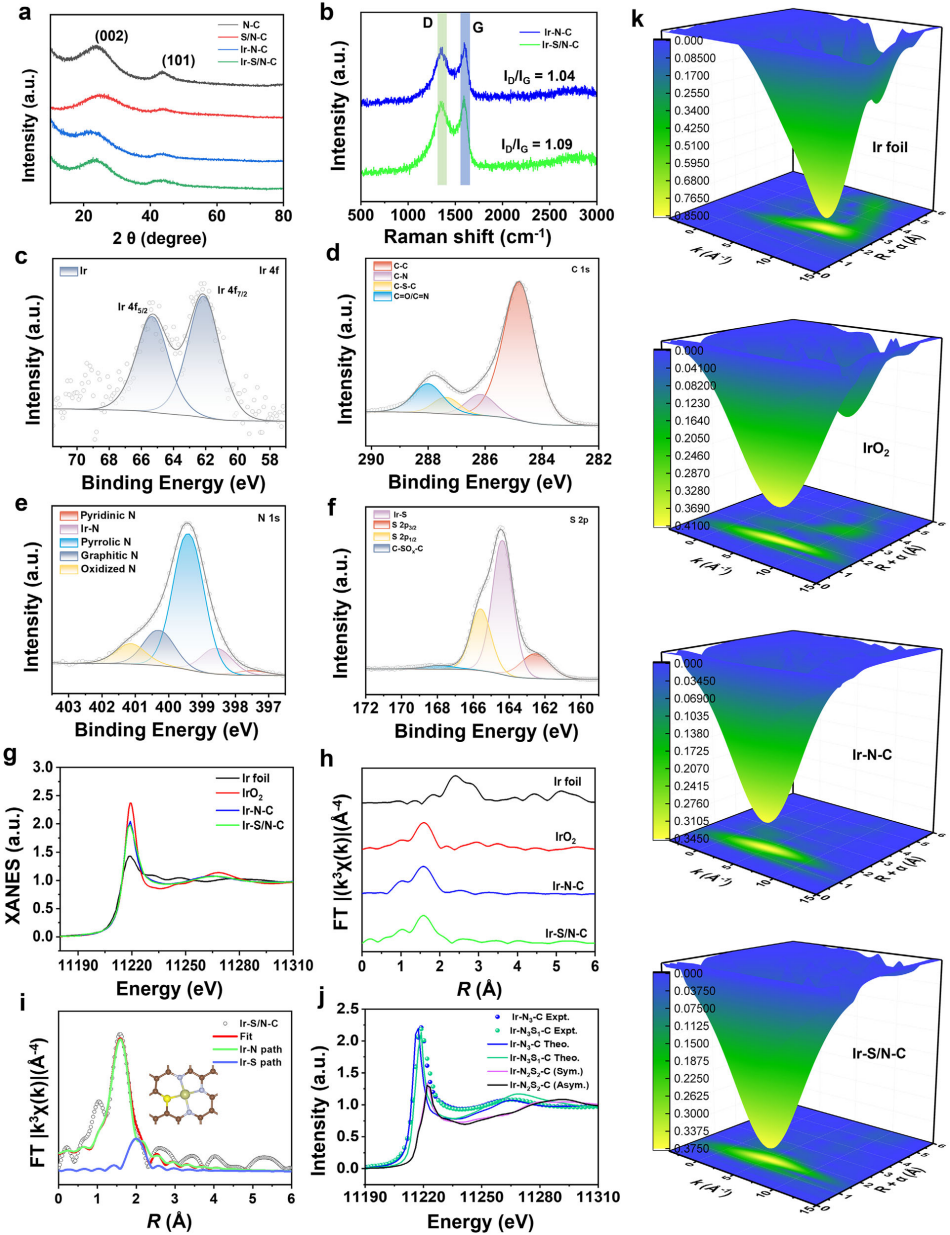
Fine structural characterization of the Ir–S/N–C SAzymes. (a) XRD patterns of Ir–S/N–C and the N–C, S/N–C, and Ir–N–C control samples. (b) Raman spectra showing the D and G‐bands of the Ir–N–C and Ir–S/N–C SAzymes. High‐resolution XP spectra of the (c) Ir 4f, (d) C 1s, (e) N 1s, and (f) S 2p orbitals of the Ir–S/N–C SAzymes. (g) Ir L_3_‐edge XANES spectra of Ir–S/N–C and the Ir foil, IrO_2_, and Ir–N–C reference samples. (h) Fourier‐transformed (FT) EXAFS spectra showing the local atomic environment of Ir in different samples. (i) k^3^‐weighted EXAFS fitting curves in R‐space for the Ir–S/N–C SAzymes: inset: optimized Ir–N_3_S_1_ coordination model. (j) Corresponding theoretical XANES spectra calculated using FDMNES code for Ir–N_4_, Ir–N_3_, Ir–N_3_S_1_, Ir–N_2_S_2_ (asymmetric), and Ir–N_2_S_2_ (symmetric). (k) WT of the Ir L_3_‐edge EXAFS signals for Ir–S/N–C and the Ir foil, IrO_2_, and Ir–N–C reference samples.

To better understand the local atomic environment and coordination structure of Ir in the Ir–S/N–C SAzyme, its structure was comprehensively characterized. The XRD pattern displayed only two broad peaks at approximately 24° and 43°, corresponding to the (002) and (101) planes of graphitic carbon [33], respectively, thus confirming the presence of an amorphous or a disordered carbon framework and the absence of Ir‐related crystalline phases (Figure 2a). Raman spectroscopy was conducted to determine the structural details of the carbon matrix. As shown in Figure 2b, distinct peaks appeared at 1350 cm^−1^ (D‐band) and 1580 cm^−1^ (G‐band), attributed to disordered and sp^2^‐hybridized graphitic carbon, respectively. The intensity ratio of these two bands (I_D_/I_G_) for Ir–S/N–C was calculated to be 1.09, which was higher than that of Ir–N–C (1.04), indicating a higher degree of structural disorder and defect density. This increase in defects was likely due to S doping and high‐temperature pyrolysis, which introduced more edge sites and distorted regions [16, 34, 35]. These defects were beneficial because they could serve as additional anchoring sites for Ir atoms and may also have enhanced the catalytic activity by facilitating mass and charge transfer.

The coordination environments and surface chemical states were determined through x‐ray photoelectron (XP) spectroscopy (XPS). In the Ir 4f region, Ir–S/N–C exhibited two characteristic peaks located at approximately 62.1 and 65.3 eV, assigned to Ir 4f_7/2_ and Ir 4f_5/2_, respectively. No signal corresponding to metallic Ir^0^ was detected, confirming that single Ir atoms were successfully dispersed (Figure 2c). Relative to those of Ir–N–C, the Ir 4f peaks of Ir–S/N–C shifted toward a more positive binding energy (Figure S9), implying that S incorporation induced a stronger electron‐withdrawing effect [36], which suggested the formation of an Ir–N_x_S_y_ coordination environment. In the C 1s spectra, Ir–S/N–C displayed four main peaks centered at approximately 284.7, 286.1, 287.3, and 288.0 eV, corresponding to the C–C, C–N, C–S–C, and C═O/C═N species, respectively (Figure 2d). The high‐resolution N 1s spectrum of Ir–S/N–C was deconvoluted into pyridinic N (397.5 eV), pyrrolic N (399.4 eV), graphitic N (400.3 eV), oxidized N (401.1 eV), and Ir–N (398.5 eV) components, confirming N species were key coordination ligands for single Ir atoms (Figure 2e). These N species, particularly pyridinic and Ir–N, act as electron donors, facilitating charge redistribution around metal centers [37]. Meanwhile, the S 2p spectrum displayed characteristic doublet peaks at 164.4 and 165.6 eV, corresponding to thiophene‐like S species that enhanced the electronic conductivity of the carbon matrix (Figure 2f). Importantly, the peak at 162.5 eV was assigned to Ir─S bonding, directly demonstrating that S atoms helped stabilize the Ir single atoms. A minor feature at 167.9 eV indicated the presence of oxidized S (C–SO_x_–C), which may have provided additional surface reactivity [38]. By contrast, no S 2p peak appeared for Ir–N–C, which confirmed that S was successfully incorporated into the framework (Figure S10). The XPS results collectively demonstrated that the Ir atoms in Ir–S/N–C were asymmetrically coordinated by N and S, forming well‐defined Ir–N_x_S_y_ active sites. This dual‐ligand coordination not only stabilized the atomically dispersed Ir centers but also modulated their local electronic structure through synergistic interactions among Ir, N, and S.

To elucidate the local coordination environment of Ir in the I–S/N–C SAzyme, Ir L_3_‐edge x‐ray absorption spectroscopy was conducted. As shown in the XANES spectra (Figure 2g), the absorption edges of both Ir–S/N–C and Ir–N–C lay between those of the metallic Ir foil and IrO_2_, indicating that the Ir species in both SAzymes adopted an intermediate oxidation state between 0 and +4. This observation, which was consistent with the XPS results, suggested that the Ir atoms were coordinated with electronegative heteroatoms (N and S) and that metallic Ir^0^ was absent. The corresponding Fourier‐transformed extended x‐ray absorption fine structure (EXAFS) spectra (Figure 2h) revealed the absence of characteristic Ir–Ir scattering peaks in both Ir–S/N–C and Ir–N–C, confirming the atomically dispersed state of Ir. Notably, the dominant peak centered at ∼1.56 Å in both samples was attributed to the coordination of Ir with light atoms (N and S) in the first coordination shell. Detailed EXAFS fitting (Figure S11 and Figure 2i) revealed that Ir in Ir–S/N–C was coordinated with three N atoms and one S atom, forming an asymmetric Ir–N_3_S_1_ structure with average Ir─N and Ir─S bond lengths of 2.00 and 2.51 Å, respectively (Table S1). By contrast, Ir in Ir–N–C adopted a more symmetric Ir–N_3_ coordination configuration with an Ir─N bond length of 2.01 Å (Figure S12 and Table S1).

To further verify the structural assignment and exclude alternative coordination models, we constructed five representative structural models: Ir–N_4_, Ir–N_3_, Ir–N_3_S_1_, Ir–N_2_S_2_ (asymmetric), and Ir–N_2_S_2_ (symmetric), which were optimized using DFT before calculating their theoretical XANES spectra using FDMNES code [39]. This model comparison strategy is consistent with the standard use of theoretical XANES simulations to discriminate local coordination environments in single‐atom catalysts. The simulated XANES spectrum of the Ir–N_3_ model more closely matched the experimental spectrum of the Ir–N control sample than that of the Ir–N_4_ model, confirming that the control sample adopted the Ir–N_3_ configuration rather than Ir–N_4_ (Figure S13a,b). For the target sample, the Ir–N_3_S_1_ model showed the best agreement with the experimental XANES features, whereas both the symmetric and asymmetric Ir–N_2_S_2_ models deviated markedly, supporting the assignment of the Ir–N_3_S_1_ coordination structure and ruling out higher S‐coordination alternatives (Figure 2j, Figure S13c–e).

Further insights into the coordination symmetry were obtained from a wavelet transform (WT) analysis (Figure 2k). The WT contour of Ir–S/N–C exhibited a dominant intensity maximum centered at R = 1.56 Å and *k* = 6–8 Å^−1^, which was consistent with light‐element backscattering (Ir–N or Ir–S coordination). Relative to that of Ir–N–C, the maximum intensity of Ir–S/N–C shifted discernibly toward higher *k* values, reflecting the contribution of heavier S atoms to N coordination. By contrast, Ir foil displayed a characteristic high‐intensity feature at R = 2.4–2.6 Å and *k* = 10–12 Å^−1^, corresponding to Ir–Ir scattering, whereas IrO_2_ showed intermediate features associated with Ir–O coordination in lower *k* regions. Notably, no intensity maximum was observed in the high‐*k* (>9 Å^−1^), high‐R (>2.2 Å) region for Ir–S/N–C, quantitatively excluding Ir–Ir coordination. Collectively, these EXAFS results provide compelling evidence that Ir in Ir–S/N–C existed as isolated atoms stabilized by a unique Ir–N_3_S_1_ coordination environment. This asymmetric S coordination was expected to modulate the electronic structure of Ir, adjust its *d*‐band center, and enhance charge redistribution [40, 41], contributing to the superior multienzyme‐like catalytic activity of the Ir–S/N–C SAzyme.

### 2.3 Multienzyme‐Like Activity of Ir–S/N–C SAzymes

**FIGURE 3:**
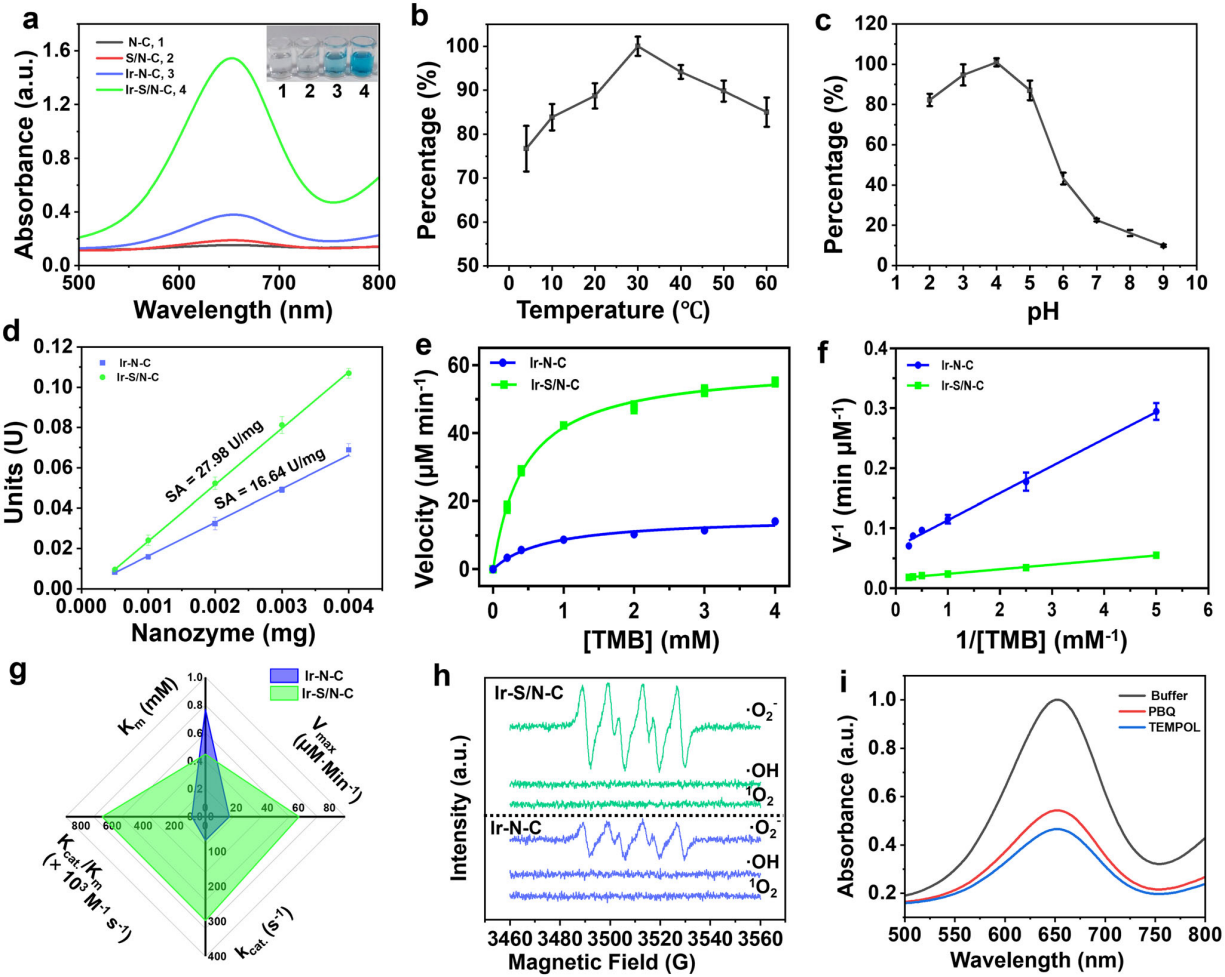
OXD‐like kinetics analysis. (a) Comparison of the UV–vis absorption curves for Ir–S/N–C and the N–C, S/N–C, and Ir–N–C reference samples; inset: photograph showing the corresponding color changes. Optimization of the OXD‐like activity of the Ir–S/N–C SAzymes at different (b) temperatures and (c) pH. (d) Specific activity of the Ir–N–C and Ir–S/N–C SAzymes. (e) Michaelis–Menten kinetics of the Ir–N–C and Ir–S/N–C SAzymes with TMB substrates and (f) the corresponding Lineweaver–Burk plots. (g) OXD‐like kinetics (K_m_, V_max_, k_cat._, and k_cat._/K_m_ values) of the Ir–N–C and Ir–S/N–C SAzymes. (h) EPR spectra recorded for •O_2_^−^, •OH, and ^1^O_2_. (i) OXD‐like catalytic activity of Ir–S/N–C in the presence of superoxide scavengers.

The enzyme‐mimetic properties of the Ir–S/N–C SAzymes were systematically investigated, focusing on their OXD‐, POD‐, and GSHOx‐like activities. Figure 3a shows that Ir–S/N–C had the highest catalytic activity toward the oxidation of 3,3',5,5'‐tetramethylbenzidine (TMB) in the absence of H_2_O_2_, outperforming Ir–N–C, S/N–C, and N–C. Figure 3b shows the temperature dependence of the OXD activity, indicating that both Ir–S/N–C and Ir–N–C performed optimally at 30°C. Similarly, Figure 3c demonstrates that the activity depended on the pH, peaking under acidic conditions (pH 3–4), which was consistent with the behavior of many OXD‐mimicking nanozymes [42, 43]. Time‐dependent UV–vis absorbance measurements at 652 nm (Figure S14a,b) indicated that Ir–S/N–C catalyzed the TMB oxidation reaction more rapidly than Ir–N–C, reaching saturation within a shorter time. The corresponding specific activity of Ir–S/N–C was calculated to be 27.98 U mg^−1^,1.68 times higher than that of Ir–N–C (16.64 U mg^−1^; Figure 3d), validating its superior catalytic efficiency. Kinetic investigations using the Michaelis–Menten model also indicated an increase in enzyme‐like activity (Figure 3e–g). Ir–S/N–C exhibited a higher V_max_ (60.23 µm min^−1^) and lower K_m_ (0.4470 mm) than Ir–N–C (V_max_ = 15.38  µm  min^−1^; K_m_ = 0.7674 mm), indicating a stronger substrate affinity and faster catalytic turnover. The calculated turnover number (k_cat._) of Ir–S/N–C reached 296.99 s^−1^, with a catalytic efficiency (k_cat._/K_m_) of 6.64 × 10^5^ m ^−1^·s^−1^, which was nearly 7.56 times higher than that of Ir–N–C (87.91 × 10^3^ m ^−1^·s^−1^). These findings clearly show that introducing S into the Ir–N coordination environment improved the substrate interactions while accelerating the reaction kinetics, thus increasing the OXD‐like activity.

To further verify the catalytic mechanism of Ir–S/N–C, thiocyanate ions (SCN^−^) were used to poison the Ir active sites. SCN^−^ treatment markedly suppressed the OXD‐like activity of Ir–S/N–Cter, indicating that the catalytic activity predominantly originated from atomically dispersed Ir centers (Figure S15a). In contrast, ethylene diamine tetraacetic acid (EDTA) did not significantly affect this activity, suggesting the catalytic process relied on stable Ir sites within the carbon matrix rather than free metal ions. Moreover, the OXD‐like activity was significantly enhanced under O_2_‐saturated conditions, suggesting that dissolved oxygen directly participated in the catalytic oxidation process as an electron acceptor (Figure S15b). These results imply that Ir–S/N–C efficiently activated O_2_ to generate reactive oxygen species (ROS) for TMB oxidation.

Next, electron paramagnetic resonance (EPR) spectroscopy was performed to identify the reactive intermediates generated during catalysis. Figure 3h clearly exhibits the characteristic signals of superoxide radicals (•O_2_^−^), whereas no signals associated with hydroxyl radicals (•OH) or singlet oxygen (^1^O_2_) appear, indicating that the catalytic pathway mainly depended on •O_2_^−^ generation. Notably, Ir–S/N–C produced substantially stronger •O_2_^−^ signals than Ir–N–C, demonstrating that asymmetric Ir–N_3_S_1_ coordination facilitated oxygen activation and promoted •O_2_^−^ generation. To verify the dominant role of •O_2_^−^, 2,2,6,6‐tetramethyl‐1‐piperidinyloxyl (TEMPOL) and *p*‐benzoquinone (PBQ), two typical superoxide scavengers, were introduced into the catalytic system [44]. As shown in Figure 3i, both scavengers significantly inhibited the OXD‐like activity of Ir–S/N–C, confirming that •O_2_^−^ served as the primary reactive species during the OXD‐like catalytic process. Additionally, morphologically uniform Ir nanoparticles (Ir NPs) were prepared (Figure S16). Compared with both Ir–N–C and Ir NPs, Ir–S/N–C exhibited higher OXD‐like activity (Figure S17a). The EPR results further demonstrated its superior ability to generate •O_2_^−^ (Figure S17b). Collectively, these results demonstrate that the asymmetric Ir–N_3_S_1_ coordination environment enhanced O_2_ adsorption and activation at atomic Ir sites, promoting •O_2_^−^‐mediated OXD‐like catalysis.

**FIGURE 4:**
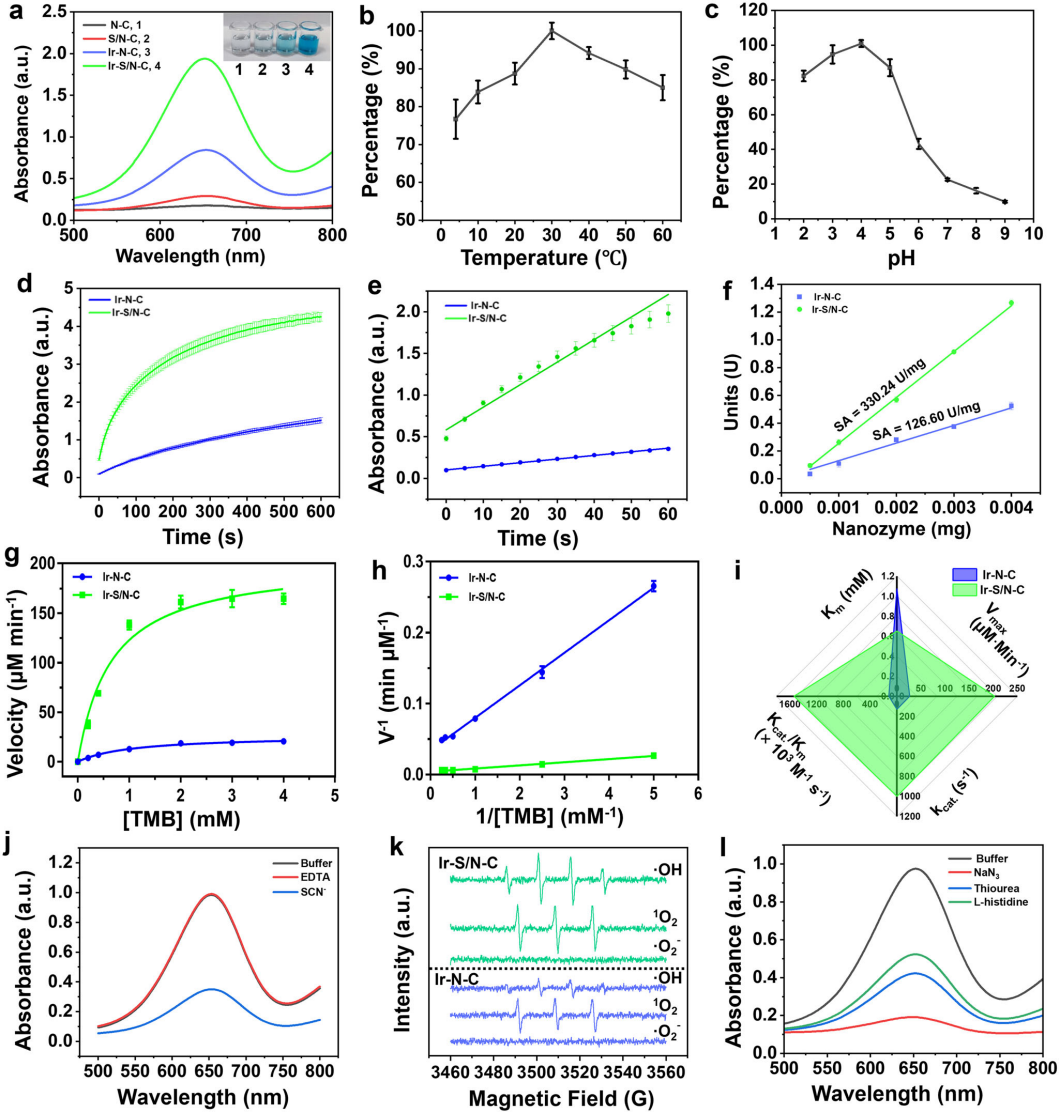
POD‐like kinetics analysis. (a) Comparison of the UV–vis absorption curves for Ir–S/N–C and the N–C, S/N–C, and Ir–N–C reference samples. Insets: photographs displaying the corresponding color changes. Optimization of the POD‐like activity of the Ir–S/N–C SAzymes at different (b) temperatures and (c) pH. (d) Reaction–time curves at 652 nm catalyzed by the Ir–N–C and Ir–S/N–C SAzymes with the substrate of TMB and H_2_O_2_. (e) Magnified view of the initial linear portion of the reaction–time curves. (f) Specific activity of the Ir–N–C and Ir–S/N–C SAzymes. (g) Michaelis–Menten kinetics of the Ir–N–C and Ir–S/N–C SAzymes with TMB substrates, and (h) the corresponding Lineweaver–Burk plots. (i) Comparison of the POD‐like kinetics (K_m_, V_max_, k_cat._, and k_cat._/K_m_ values) of the Ir–N–C and Ir–S/N–C SAzymes with TMB substrates. (j) POD‐like catalytic activity of Ir–S/N–C before and after SCN^−^ poisoning. (k) EPR spectra recorded for •O_2_^−^, •OH and ^1^O_2_. (l) POD‐like catalytic activity of Ir–S/N–C in the presence of •OH and ^1^O_2_ scavengers.

The POD activity of the Ir–S/N–C SAzymes was evaluated using TMB and H_2_O_2_ as substrates. As demonstrated in Figure 4a, Ir–S/N–C had a much higher POD activity than Ir–N–C, S/N–C, and N–C, highlighting the importance of asymmetric Ir–N_3_S_1_ coordination to the catalytic function. Figure 4b,c indicate that both Ir–S/N–C and Ir–N–C exhibited maximum activity at 30°C and under acidic conditions (pH 3–4), consistent with the properties of typical POD‐mimicking nanozymes [45, 46]. Time‐resolved monitoring of the reaction at 652 nm (Figure 4d, e) showed that Ir–S/N–C catalyzed the TMB–H_2_O_2_ reaction more rapidly than Ir–N–C and thus reached saturation earlier, reflecting faster substrate turnover. The specific activity of Ir–S/N–C was calculated to be 330.24 U mg^−1^, which was 2.61 times that of Ir–N–C (126.60 U mg^−1^; Figure 4f), confirming its superior catalytic efficiency.

To quantify the catalytic parameters, Michaelis–Menten kinetic analysis was performed using TMB and H_2_O_2_ as substrates. For TMB (Figure 4g–i), Ir–S/N–C exhibited a higher V_max_ (202.70  µm·min^−1^) and k_cat._ (999.51 s^−1^) and a lower K_m_ (0.6501 mm) than Ir–N–C (V_max_ = 26.54  µm·min^−1^, k_cat._ = 130.87 s^−1^, K_m_ = 1.072 mm). The catalytic efficiency (k_cat._/K_m_) of Ir–S/N–C reached 1.54 × 10^6^ m ^−1^·s^−1^, which was over 12.6 times higher than that of Ir–N–C (1.22 × 10^5^ m ^−1^·s^−1^). Similarly, when H_2_O_2_ was used as the substrate (Figure S18a–c), Ir–S/N–C again outperformed Ir–N–C, exhibiting a V_max_ of 942.9  µm·min^−1^, k_cat._ of 4649.41 s^−1^, K_m_ of 31.21 mm, and k_cat._/K_m_ of 1.49 × 10^5^ m ^−1^·s^−1^. By contrast, Ir–N–C exhibited a significantly lower k_cat._ (604.04 s^−1^) and higher K_m_ (48.23 mm), resulting in a k_cat._/K_m_ of only 1.25 × 10^4^ m ^−1^·s^−1^. These findings show that introducing S into the Ir coordination environment significantly improved the substrate affinity and turnover rates through the formation of an asymmetric Ir–N_3_S_1_ configuration that tuned the electronic structure of the active center and optimized TMB and H_2_O_2_ adsorption.

To further elucidate the POD‐like catalytic mechanism of Ir–S/N–C, SCN^−^ was employed to poison the Ir active sites, which drastically suppressed POD activity, confirming that the atomically dispersed Ir centers were the main catalyst (Figure 4j). Similarly, EDTA did not significantly affect the catalytic activity, further demonstrating that the catalytic process relied on the stable, atomically dispersed Ir sites within the carbon framework rather than free metal ions in solution. In the conventional POD‐like pathway of nanozymes, H_2_O_2_ activation generates highly reactive hydroxyl species that oxidize TMB. Indeed, the EPR spectra contained distinct •OH and ^1^O_2_ signals, whereas no •O_2_^−^ signal was appreciable, indicating that POD‐like oxidation was dominated by •OH/^1^O_2_‐mediated pathways (Figure 4k). Notably, Ir–S/N–C produced stronger radical signals than Ir–N–C. Accordingly, sodium azide (NaN_3_) was employed to scavenge both •OH and ^1^O_2_, L‐histidine was used to quench ^1^O_2_, thiourea was used as an •OH scavenger, and TMB was used as a chromogenic substrate [47]. The scavenger assays consistently showed that NaN_3_, L‐histidine, and thiourea markedly attenuated the UV–vis absorption peak of oxidized TMB at 650 nm, with NaN_3_ showing the strongest inhibition, further validating that •OH and ^1^O_2_ participated in the POD‐like process (Figure 4l). Additionally, the POD‐like activity was compared with that of Ir NPs, and Ir–S/N–C exhibited significantly higher enzymatic activity (Figure S19a). The EPR results further demonstrated its enhanced capability for •OH generation (Figure S19b). Collectively, these results demonstrate that asymmetric Ir–N_3_S_1_ coordination promoted H_2_O_2_ activation and the formation of ROS intermediates, accounting for the superior POD‐like activity of the Ir–S/N–C SAzymes.

Next, the GSHOx‐like activities of Ir–S/N–C and Ir–N–C were evaluated using 5,5'‐dithiobis‐(2‐nitrobenzoic acid) (DTNB) as the probe. As shown in Figure S20a, the UV–vis absorption changed negligibly with N–C and S/N–C, whereas Ir–N–C and Ir–S/N–C exhibited pronounced decreases in the intensity of the the peak at 412 nm, indicating the efficient oxidation of GSH. Time‐dependent studies (Figure S20b, c) revealed that Ir–S/N–C consumed GSH much more rapidly (within 5 min) than Ir–N–C, highlighting its superior catalytic kinetics. Moreover, atmosphere‐dependent experiments (Figure S20d) demonstrated enhanced activity under O_2_, moderate activity in air, and suppressed performance under N_2_, thus confirming that O_2_ adsorption and activation played critical roles in the catalytic process [48]. Collectively, these findings demonstrate that asymmetric Ir–N_3_S_1_ coordination stabilized the atomically distributed Ir while tailoring the local electronic structure, maximizing substrate affinity, accelerating turnover, and increasing the multienzyme mimic activity.

To further evaluate the electron‐transfer capabilities of the catalysts, cytochrome c (Cyt C) was employed as an electron acceptor [49]. As shown in Figure S21a, the Cyt C + Ir–S/N–C group exhibited a much more pronounced decrease in the characteristic UV–vis absorption band at 550 nm than Cyt C alone and Cyt C + Ir–N–C, indicating more efficient electron transfer between Ir–S/N–C and Cyt C. Further, electrochemical impedance spectroscopy (EIS) was performed to investigate the interfacial charge‐transfer behavior [49]. As shown in Figure S21b, Ir–S/N–C displayed a substantially smaller Nyquist semicircle diameter than Ir–N–C, implying a lower charge‐transfer resistance and faster interfacial electron‐transport kinetics. Taken together, these results confirm that asymmetric Ir–N_3_S_1_ coordination facilitated electron redistribution and accelerated electron‐transfer processes, thereby improving the multienzyme‐like catalytic activity of Ir–S/N–C.

The stability and durability of the Ir–S/N–C SAzyme were also evaluated to assess its potential in practical applications. As shown in Figure S22a–c, Ir–S/N–C retained a high proportion of its OXD, POD, and GSHOx activities after 60 days of storage, demonstrating excellent preservaability. Recycling experiments revealed that the catalytic activity remained nearly unchanged after six reaction cycles, highlighting the remarkable reusability of the Ir–S/N–C SAzyme (Figure S23a–c). Structural characterization provided additional evidence for this robustness. In detail, the TEM and SEM images (Figure S24) confirmed that the overall morphology of Ir–S/N–C was well preserved after repeated catalytic reactions, with no obvious aggregation or collapse. EDS mapping (Figure S25) showed that C, N, S, and Ir remained homogeneously distributed, whereas the XRD pattern (Figure S26) displayed no new diffraction peaks, indicating that the amorphous or disordered carbon framework structure remained stable during catalytic cycling. Furthermore, the reaction solution was collected after centrifugation and analyzed by ICP–OES, and no Ir species were detectable in the supernatant, confirming that the Ir single atoms were firmly stabilized within the coordination framework, with no observable metal leaching during catalysis. Collectively, these results demonstrate that Ir–S/N–C possessed excellent structural stability and catalytic robustness during storage and repeated use, highlighting its good prospects for applications in biocatalysis, environmental monitoring, and biomedical diagnostics.

### 2.4 DFT Calculation Studies on Multienzyme‐Like Activity

**FIGURE 5:**
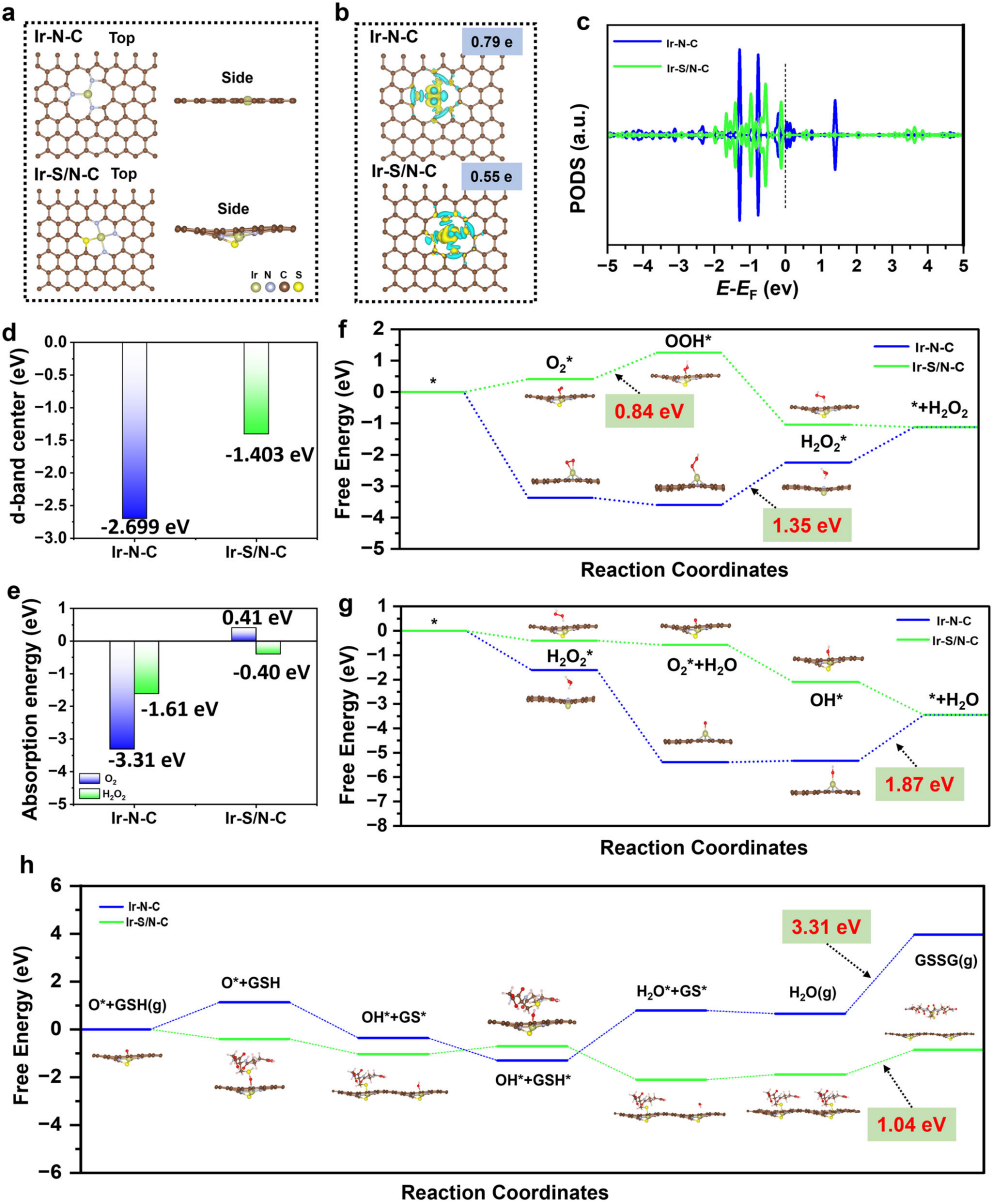
DFT calculations and structural models of the Ir–N–C and Ir–S/N–C SAzymes. (a) Optimized atomic models of the Ir–N–C and Ir–S/N–C configurations viewed from the top and side. (b) Charge density difference maps of the Ir–N–C and Ir–S/N–C models, showing regions of electron accumulation and depletion. (c) PDOS of Ir–N–C and Ir–S/N–C. (d) Calculated *d*‐band center positions for Ir–N–C and Ir–S/N–C. (e) Adsorption energies of O_2_^*^ and H_2_O_2_^*^ intermediates on the Ir–N–C and Ir–S/N–C surfaces. Free energy diagrams of the catalytic reaction pathways: (f) OXD‐like, (g) POD‐like, and (h) GSHOx‐like reactions on the Ir–N–C and Ir–S/N–C models.

To determine the origin of the improved OXD, POD, and GSHOx‐like activities of Ir–S/N–C, DFT calculations were performed, and the results were compared with those of Ir–N–C. The optimized atomic models revealed that Ir–S/N–C had an asymmetric Ir–N_3_S_1_ coordination geometry, unlike the symmetric Ir–N_3_‐like environment in Ir–N–C (Figure 5a). Changing the coordination environment redistributes the electronic structure at the active site. A Bader charge analysis showed that the Ir center in Ir–S/N–C and Ir–N–C lost 0.55 and 0.79 e, respectively, indicating more moderate electron donation in the Ir–S/N–C system (Figure 5b) [44]. Varying the coordination environment also affects the electronic energy levels. A partial density of states (PDOS) analysis (Figure 5c) revealed that the *d*‐band center of Ir–S/N–C (−1.403 eV) was above that of Ir–N–C (−2.699 eV; Figure 5d), indicating enhanced orbital overlap and stronger electronic coupling between the Ir site and adsorbed reactants. The combined effects of modulating the charge and electronic structure were likely to jointly regulate the adsorption strength of reactant molecules, contributing to a more balanced interaction between intermediate stabilization and product desorption. Adsorption energy calculations (Figure 5e) confirmed that Ir–S/N–C binds O_2_ and H_2_O_2_ (0.41 and −0.40 eV, respectively) more weakly than Ir–N–C (1.61 and −3.31), preventing overbinding and allowing for faster catalytic turnover.

The DFT calculations revealed that the OXD‐like activity of Ir–S/N–C stemmed from its capacity to lower the energy barrier of the rate‐determining step (RDS; Figure 5f). The catalytic cycle began with O_2_ adsorption at the Ir center, followed by proton–electron transfer to form OOH^*^. On Ir–S/N–C, this O_2_^*^ → OOH^*^ step required only 0.84 eV. By contrast, on Ir–N–C, the RDS was the subsequent OOH^*^ → H_2_O_2_ step, with a barrier of 1.35 eV. The 0.51 eV lower barrier on Ir–S/N–C arose from the product binding more weakly onto the asymmetric Ir–N_3_S_1_ site, avoiding the overbinding‐induced desorption penalty observed on Ir–N–C. According to the Arrhenius equation, a barrier reduction of 0.51 eV corresponds to a theoretical rate enhancement of 10^8^‐fold at room temperature (assuming a typical prefactor) [50]. This explains the experimentally observed faster O_2_ activation and TMB oxidation kinetics for Ir–S/N–C.

In the POD‐like pathway, H_2_O_2_ first adsorbed onto the Ir site and cleaves to generate reactive OH^*^ intermediates, which subsequently oxidized TMB (Figure 5g). Every elementary step with Ir–S/N–C was exergonic, from H_2_O_2_ adsorption to OH^*^ production and finally H_2_O desorption, resulting in a spontaneous and continuous catalytic cycle. By contrast, Ir–N–C had a large endothermic penalty (1.87 eV) for the final step of H_2_O desorption, again owing to the overbinding of the oxygenated species. This 1.87 eV vs the exergonic (negative barrier) difference indicated that H_2_O desorption was thermodynamically prohibited on Ir–N–C under catalytic conditions, directly slowing the turnover. These theoretical results were consistent with the experiments demonstrating that Ir–S/N–C had a 12.6‐fold higher catalytic efficiency than Ir–N–C in POD processes.

The GSH oxidation process proceeded through sequential adsorption and activation steps (Figure 5h). First, O_2_ bound to the Ir center and was activated to form O^*^. Upon adsorption, GSH underwent proton–electron transfer to form GS^*^ and OH^*^, followed by coupling to generate surface‐bound GS–OH intermediates. The cycle concluded with H_2_O release and the dimerization of GS^*^ to yield GSSG [48]. On Ir–N–C, the final step of H_2_O desorption and GSSG production required 3.31 eV, severely restricting its activity. By contrast, Ir–S/N–C lowered the barrier to 1.04 eV, a reduction of 2.27 eV. Such a large energy difference translated into orders‐of‐magnitude faster product release and catalyst renewal. This mechanistic advantage was consistent with the DTNB experiments, which indicated that Ir–S/N–C rapidly depleted GSH within minutes, whereas Ir–N–C took much longer. Overall, the asymmetric Ir–N_3_S_1_ coordination in Ir–S/N–C modulated the electronic structure to achieve a balanced interaction with the reaction intermediates, stabilizing the intermediates (O_2_^*^, OOH^*^, OH^*^, and GS^*^) but not excessively binding the products (H_2_O, H_2_O_2_, and GSSG). This optimized adsorption–desorption behavior reduced the energy barriers and accelerated turnover across the OXD, POD, and GSHOx pathways, in complete agreement with the experimental findings. Overall, the DFT studies confirmed that S incorporation is a potent method for increasing the multienzyme‐like activity of the Ir–S/N–C SAzymes.

To elucidate the coordination‐dependent catalytic mechanism, in situ attenuated total reflectance surface‐enhanced infrared absorption spectroscopy (ATR–SEIRAS) was performed to monitor the dynamic evolution of surface intermediates during both the OXD‐like and POD‐like catalytic processes [36]. During the OXD‐like reaction under O_2_‐saturated conditions, Ir–S/N–C exhibited much stronger and more persistent IR responses than Ir–N–C (Figure S27). In particular, Ir–S/N–C gave rise to a pronounced band at 1200–1280 cm^−1^, which could be assigned to adsorbed OOH^*^ intermediates generated during oxygen activation. By contrast, the corresponding signal for Ir–N–C was much weaker, indicating less efficient O_2_ adsorption and activation. In situ ATR–SEIRAS was further employed to probe the POD‐like catalytic pathway of Ir–S/N–C during H_2_O_2_ activation (Figure S28). Upon reaction, H_2_O_2_ gradually decomposed on the Ir–S/N–C surface to generate reactive •OH intermediates accompanied by H_2_O consumption. Meanwhile, the spectra of Ir–S/N–C evolved in a much more drastic, time‐dependent manner than that of Ir–N–C in the 1600–1720 cm^−1^ (H_2_O) and 1060–1140 cm^−1^ (OH^*^) regions, indicating enhanced H_2_O_2_ adsorption and peroxide‐intermediate formation. The kinetic advantage introduced by S coordination was further quantified by temperature‐dependent catalytic measurements. Arrhenius analysis showed that the apparent activation energy (E_a_) of the OXD‐like reaction decreased from 52 to 31 kJ mol^−^^1^ after sulfur incorporation, while that of the POD‐like reaction was reduced from 42 to 24 kJ mol^−^^1^ (Table S2). Although these apparent activation energies represent the overall reaction kinetics rather than individual elementary steps, they clearly demonstrate that the asymmetric Ir–N_3_S_1_ coordination lowers the overall kinetic barrier for both O_2_‐ and H_2_O_2_‐mediated catalytic pathways. Together with the DFT calculations, these operando spectroscopic and kinetic results consistently demonstrate that asymmetric S coordination tailors the local electronic structure of the isolated Ir center, strengthens its interaction with oxygen‐containing reactants, facilitates the evolution of key oxygenated intermediates, and reduces the overall activation barrier. This coordination‐dependent modulation of reactant activation and interfacial reaction kinetics accounts for the enhanced multienzyme‐like catalytic performance of the Ir–S/N–C SAzyme.

### 2.5 Colorimetric Analysis Using Ir–S/N–C SAzymes

**FIGURE 6:**
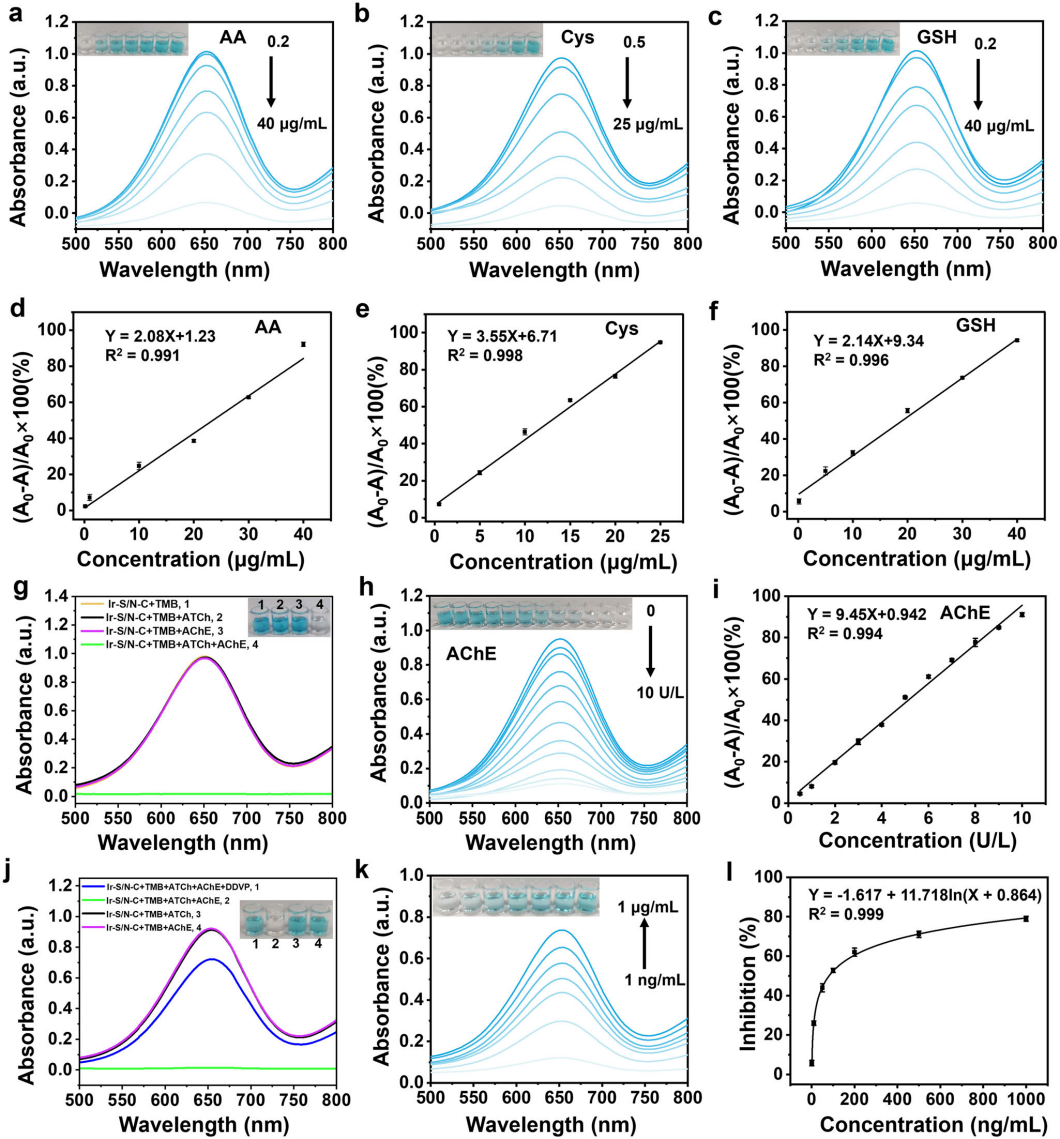
Colorimetric detection of antioxidants, AChE, and DDVP using the Ir–S/N–C SAzymes. UV–vis absorption spectra of Ir–S/N–C toward (a) AA, (b) Cys, and (c) GSH at various concentrations. (d–f) Corresponding calibration plots for AA, Cys, and GSH detection. (g) UV–vis absorption spectra showing the colorimetric response of Ir–S/N–C to AChE. (h) UV–vis absorption spectra of Ir–S/N–C with various AChE concentrations. (i) Relative absorbance as a function of the AChE concentration. (j) UV–vis absorption spectra showing Ir–S/N–C response to AChE in the presence of DDVP. (k) Absorption spectra of Ir–S/N–C at various DDVP concentrations. (l) Calibration curve showing the relationship between the inhibition percentage and DDVP concentration. Insets: photographs of the corresponding color changes in the reaction solutions.

Having established its OXD‐, POD‐, and GSHOx‐like activities, the Ir–S/N–C SAzyme was further utilized for versatile colorimetric biosensing. Owing to its robust OXD‐like activity, Ir–S/N–C enabled H_2_O_2_‐free assays of various biologically relevant molecules, including antioxidants and thiol‐containing compounds. First, the Ir–S/N–C SAzyme enabled highly sensitive colorimetric detection of AA, Cys, and GSH. As shown in Figure 6a–c, the absorbance intensity at 652 nm steadily decreased with the increasing analyte concentration, accompanied by an apparent color change from blue to nearly colorless. Quantitative calibration curves (Figure 6d–f) revealed good linear relationships among AA (Y = 2.08X + 1.23, R^2^ = 0.991), Cys (Y = 3.55X + 6.71, R^2^ = 0.998), and GSH (Y = 2.14X + 9.34, R^2^ = 0.996). The corresponding limits of detection (LODs) were estimated to be 0.32, 0.41, and 0.37 µm for AA, Cys, and GSH, respectively, indicating the high sensitivity of Ir–S/N–C toward these biologically relevant molecules. Therefore, Ir–S/N–C provided a reliable and efficient platform for detecting antioxidants and thiols, with sensitivities comparable to or exceeding those of most previously described nanozyme systems [51, 52, 53, 54, 55, 56].

In addition to small‐molecule sensing, Ir–S/N–C was employed for the colorimetric detection of AChE, a critical enzyme in nerve signal transmission and a key biomarker for pesticide exposure and neurodegenerative diseases [57, 58]. The detection strategy was based on the hydrolysis of acetylthiocholine (ATCh) by AChE to generate thiocholine, which scavenged the ROS produced by Ir–S/N–C, inhibiting TMB oxidation. As shown in Figure 6g, the control systems (Ir–S/N–C + TMB, Ir–S/N–C + TMB + AChE, and Ir–S/N–C + TMB + ATCh) produced a strong blue color with a characteristic absorption peak at 652 nm. By contrast, when ATCh and AChE were both present, the absorbance at 652 nm reduced drastically and the color faded, demonstrating that Ir–S/N–C efficiently suppressed TMB oxidation. To evaluate the sensitivity of this sensing system, various concentrations of AChE (0–10 U·L^−1^) were introduced (Figure 6h). The absorbance at 652 nm decreased progressively with increasing AChE concentration, and the signal inhibition showed a linear relationship with the enzyme concentration (Figure 6i). The calibration curve followed the regression equation Y = 9.45X + 0.942, with an R^2^ of 0.994 and LOD of 0.21 U·L^−1^. Additionally, the conventional gold‐standard method for analyzing AChE activity was employed as an independent reference assay (Figure S29). The AChE activities determined by the two methods were in good agreement, further confirming the accuracy and reliability of the developed sensing strategy. Notably, unlike traditional nanozyme‐based AChE assays that require exogenous H_2_O_2_ for POD‐like catalysis [59, 60, 61, 62], the Ir–S/N–C system depended entirely on intrinsic OXD activity with dissolved O_2_ as the oxidant. The test process was therefore simplified by this H_2_O_2_‐free sensing approach, which also improved the stability by removing H_2_O_2_‐related instability.

Building on this concept, the Ir–S/N–C SAzyme was employed for the colorimetric detection of OPs based on AChE inhibition. As shown in Figure 6j, Ir–S/N–C was colorless in the presence of ATCh and AChE. However, adding dichlorvos (DDVP), a typical OP, drastically increased the UV–vis absorbance intensity at 652 nm, indicating that DDVP hindered the AChE activity and suppressed the breakdown of ATCh into thiocholine. Consequently, fewer reducing species were available to quench the reactive oxygen intermediates produced by Ir–S/N–C, thus increasing TMB oxidation. The absorbance change was quantitatively correlated with the DDVP concentration (Figure 6k,l), showing a clear logarithmic relationship from 1 ng·mL^−1^ to 1 µg·mL^−1^, described by the regression equation Y = −1.617 + 11.718 ln(X + 0.864) (R^2^ = 0.999). The calculated LOD was as low as 0.85 ng·mL^−1^, demonstrating the high sensitivity of the proposed sensing system. Notably, this H_2_O_2_‐independent sensing mechanism imparted higher operational stability and eliminated POD‐related artifacts; thus, the Ir–S/N–C SAzyme outperformed conventional Fe–N–C or Ce–N–C nanozymes, which rely on external H_2_O_2_ for POD‐like catalysis (Table S3) [63, 64]. Moreover, Ir–S/N–C exhibited excellent selectivity toward AChE‐targeting OPs, because only OP compounds such as trichlorfon and chlorpyrifos induced significant signal inhibition (>95%). By contrast, non‐OP pesticides, including chlorfenapyr, indoxacarb, tebufenozide, lufenuron, and flufiprole, as well as common inorganic ions caused negligible interference (Figures S30 and S31a). In addition, the sensing performance remained essentially unchanged in the presence of complex interfering species, including BSA, glucose, mixed cations, and mixed anions, demonstrating the strong anti‐interference capability of the Ir–S/N–C SAzyme platform in complicated matrices (Figure S31b). This finding was consistent with the intrinsic robustness of nanozyme‐based sensing systems under complex conditions [65]. The effects of pH and ionic strength on the sensing performance were further investigated. The signal deviated significantly under pH 6.0 and 8.5 conditions, which could be attributed to the pH‐dependent inhibition of AChE activity after DDVP incubation, thereby indirectly affecting the colorimetric response (Figure S32a). Similarly, elevated ionic strength also influenced the enzymatic reaction, and sensing performance decreased noticeably at NaCl concentrations of 300 mm owing to the reduced activity of AChE under high‐salt conditions (Figure S32b). Nevertheless, these interferences could be effectively minimized by adjusting the pH and ionic strength of the sample solution prior to detection, thereby ensuring reliable OP analysis in practical applications.

The practicality of the Ir–S/N–C‐SAzyme‐based colorimetric sensing system was further verified by quantitatively determining DDVP in real samples, including tap water, apples, and human whole blood. As summarized in Table 1, the proposed colorimetric assay exhibited satisfactory analytical accuracy and precision across all the tested matrices. The recoveries ranged from 91.73% to 101.99%, with relative standard deviations (RSDs) below 8%, indicating high reproducibility and minimal matrix interference. The results obtained using the Ir–S/N–C colorimetric method were in good agreement with those measured by liquid chromatography–mass spectrometry (LC–MS), which yielded recoveries of 95.83%–102.26% and RSDs of <4.2%. These findings confirm that the Ir–S/N–C‐SAzyme‐based platform provided reliable quantitative performance comparable to that of standard LC–MS analysis while offering the significant advantages of simplicity, rapidity, and reagent‐free operation. Its successful application in complex biological and food samples highlights its potential for practically monitoring OP contamination in environmental and clinical settings.

**TABLE 1:** Determination of DDVP in the real samples (*n* = 3).

Sample	Added (ng mL^−1^)	Ir–S/N–C‐based Colorimetric Sensing			LC–MS		
		Found (ng mL^−1^)	Recovery (%)	RSD (%)	Found (ng mL^−1^)	Recovery (%)	RSD (%)
Tap water	10	9.95	99.54	6.42	9.87	98.70	2.34
	100	98.17	98.17	5.95	99.23	99.23	2.49
	500	484.76	96.95	5.14	500.76	100.15	3.40
Apple	10	9.51	95.09	5.11	9.58	95.83	2.82
	100	101.99	101.99	5.87	99.13	99.13	4.10
	500	505.56	101.11	4.09	494.17	98.83	1.29
Human whole blood	10	9.17	91.73	5.04	9.90	98.97	3.62
	100	92.33	92.33	7.99	101.37	101.37	2.83
	500	491.57	98.31	5.50	511.32	102.26	2.47

Recent advances in nanozyme research have emphasized that despite remarkable progress in catalytic activity enhancement, simultaneously achieving high selectivity, mechanistic clarity, and operational stability under realistic sample conditions remains a major challenge for artificial enzyme systems [23, 66, 67]. Atomically dispersed catalytic centers with well‐defined coordination environments provide unique opportunities to bridge the gap between fundamental catalytic regulation and practical biosensing applications [68, 69]. The asymmetric Ir–N_3_S_1_ coordination structure developed in this study improved not only the multienzyme‐like catalytic activity and interfacial electron‐transfer kinetics but also the ROS evolution selectivity and substrate activation behavior, enabling amplified and reliable colorimetric detection in complicated matrices. Such coordination‐dependent electronic modulation may represent an effective strategy for addressing the long‐standing trade‐off between catalytic efficiency and sensing selectivity in SAzyme‐based systems. Furthermore, the combination of operando spectroscopy, theoretical modeling, and practical sample validation provided mechanistic insights into how the local S atom coordination governed catalytic pathways, which may facilitate the rational development of next‐generation SAzyme‐based platforms for intelligent biosensing, environmental monitoring, and clinical diagnostics.

## 3 Conclusion

This study established asymmetric N/S coordination as an effective and general strategy for regulating the electronic structure and catalytic behavior of SAzymes. By introducing S into an Ir–N–C framework, an Ir–N_3_S_1_ coordination environment was successfully constructed, which disrupted the local symmetry and induced favorable charge redistribution, markedly enhancing OXD and POD activities while imparting GSHOx functionality. Intrinsically strong oxidase activity enabled the development of a self‐sufficient, H_2_O_2_‐free colorimetric sensing platform, offering improved operational simplicity, stability, and reliability for biochemical analysis. Further, this study demonstrated high sensitivity and robustness across environmental, food, and biological samples as well as system‐specific insights into coordination‐driven activity modulation. Overall, this work demonstrates that asymmetric Ir–N_3_S_1_ coordination effectively enhanced multienzyme‐like catalysis by regulating the local electronic structure of isolated Ir sites. The findings provide a promising basis for the rational design of future coordination‐engineered single‐atom nanozymes for catalytic and biosensing applications.

## Author Contributions

**T.L**.: Conceptualization, Methodology, Funding acquisition, Writing – original draft, Investigation. **X.Z**.: Methodology, Software. **J.X**.: Methodology, Data curation. **M.W**.: Methodology, Validation. **C.L**.: Methodology, Formal analysis. **Y.S**.: Software. **W.Z**.: Validation. **M.Q**.: Methodology. **W.W**.: Validation. **W.D**.: Funding acquisition, Supervision. **S.X**.: Conceptualization, Writing – review & editing, Supervision, Funding acquisition. All the authors commented on the manuscript and have given approval to the final version.

## Conflicts of Interest

The authors declare no conflicts of interest.

## Supporting information




**Supporting File**: advs76542‐sup‐0001‐SuppMat.docx.

## Data Availability

The data that support the findings of this study are available from the corresponding author upon reasonable request.
