# Supplementary material for: Coordination‐Tuned Iridium Single‐Atom Nanozymes Boost Multienzyme Activity for Colorimetric Sensing
Source: Adv Sci (Weinh). 2026 Jul 29:e76542. Online ahead of print. doi: 10.1002/advs.76542 (PMC13418507; doi:10.1002/advs.76542)
Supplement: Supplementary file 1 — Supporting File: advs76542‐sup‐0001‐SuppMat.docx. [file ADVS-9999-e76542-s001.docx]

**Supporting Information**

**Coordination-Tuned Iridium Single-Atom Nanozymes Boost Multienzyme Activity for Colorimetric Sensing**

Tao Li^a^, Xinyu Zhang^b^, Jiashan Xia^a^, Mengyu Wu^a^, Cong Liu^a^, Yapei Sun^c^, Wanjiang Zhao^a^, Min Qian^a^, Wei Wang^a^, Weixia Duan^a^, Shangcheng Xu^c,a^*

^a^Chongqing Key Laboratory of Prevention and Treatment for Occupational Diseases and Poisoning, Chongqing Municipal Health Commission Key Laboratory for Emergency Poisoning Detection and Acute Care, The First Affiliated Hospital of Chongqing Medical and Pharmaceutical College, Chongqing 400060, China

^b^Center for Global Health, School of Public Health, Nanjing Medical University, Nanjing 211166, China.

^c^Department of Occupational and Environmental Health, School of Public Health, Chongqing Medical University, Chongqing, 400016, China

*Corresponding author.

E-mail addresses: xushangcheng@cqszfy.com (S. Xu)

**Materials and Methods**

**Chemicals and Materials**

Iridium(III) acetylacetonate (Ir(acac)_3_), zinc nitrate hexahydrate (Zn(NO_3_)_2_·6H_2_O), 2-methylimidazole, acetylthiocholine chloride (ATCh), and acetylcholinesterase (AChE, from electric eel) were purchased from Sigma-Aldrich. 3,3′,5,5′-tetramethylbenzidine (TMB), hydrogen peroxide (H_2_O_2_), glutathione (GSH), cysteine (Cys), ascorbic acid (AA), dichlorvos, trichlorfon, and chlorpyrifos were obtained from Aladdin (Shanghai, China). Thioglycolic acid (TCA) and 5,5′-dithiobis(2-nitrobenzoic acid) (DTNB) were supplied by Macklin Biochemical Co., Ltd. (Shanghai, China). All other pesticides and analytical-grade reagents were used as received without further purification. Ultrapure water (18.2 MΩ·cm) from a Millipore system was used throughout all experiments.

**Synthesis of Ir–S/N–C SAzyme**

The Ir–S/N–C SAzyme was synthesized via a three-step procedure. First, Ir-doped ZIF-8 (Ir-ZIF-8) was prepared using a host-guest strategy. Briefly, 2-methylimidazole (8 mmol) was dissolved in 15 mL of methanol (solution A), while Zn(NO_3_)_2_·6H_2_O (2 mmol) and Ir(acac)_3_ (0.1 mmol) were co-dissolved in 15 mL of methanol (solution B). Solution A was added into solution B under stirring for 10 min, followed by solvothermal treatment at 120 °C for 12 h in a Teflon-lined autoclave. The resulting precipitate was collected by centrifugation (12 000 g×5 min), washed three times with methanol, and vacuum-dried at 60 °C to yield a pale-yellow Ir-ZIF-8 precursor. Pristine ZIF-8 was synthesized under identical conditions without adding Ir(acac)_3_. Subsequently, surface modification with TCA was performed to introduce sulfur species. Specifically, 300 mg of Ir-ZIF-8 was ultrasonically dispersed in 60 mL of methanol, and a solution of TCA (30 mg in 40 mL of methanol) was added dropwise. The mixture was stirred for 6 h, centrifuged (12 000 g×5 min), and vacuum-dried at 60 °C to obtain Ir-ZIF-8@TCA. A control material (ZIF-8@TCA) was prepared in the same way using ZIF-8.

Finally, pyrolysis was carried out to obtain Ir–S/N–C. The Ir-ZIF-8@TCA precursor was placed in a crucible and heated to 920 °C under N_2_ flow at a rate of 5 °C·min^-1^, maintained for 3 h, and then cooled naturally to room temperature. The resulting carbonized product was denoted as Ir–S/N–C SAzymes. Control samples, including N–C (from ZIF-8), S/N–C (from ZIF-8@TCA), and Ir–N–C (from Ir-ZIF-8), were synthesized under the same pyrolysis conditions.

**Characterizations**

Transmission electron microscopy (TEM) and scanning electron microscopy (SEM) (JEOL, Japan) were employed to investigate particle morphology and surface features. High-resolution TEM (HRTEM) and selected-area electron diffraction (SAED) were used to examine lattice fringes and crystallinity, while high-angle annular dark-field scanning TEM (HAADF-STEM) coupled with energy-dispersive X-ray spectroscopy (EDS) provided direct visualization of atomically dispersed Ir sites and elemental mapping of C, N, S, and Ir. Aberration-corrected HAADF-STEM (AC-STEM, JEOL ARM 200F) was further applied to resolve single Ir atoms. Powder X-ray diffraction (XRD, Bruker D8 Advance, Cu Kα radiation, λ = 1.5406 Å) was conducted to confirm the crystal structure and phase purity of the precursors and pyrolyzed samples. Raman spectra were collected on a Renishaw inVia Raman microscope using a 532 nm excitation laser to evaluate graphitization degree and defect structures. X-ray photoelectron spectroscopy (XPS, Thermo Scientific ESCALAB 250Xi) was used to analyze surface composition and chemical states of C, N, S, and Ir, with binding energies calibrated to the C 1s peak at 284.8 eV. In situ attenuated total reflectance surface-enhanced infrared absorption spectroscopy (ATR-SEIRAS) measurements were conducted on a Bruker Vertex 70 Fourier-transform infrared spectrometer (Bruker, Germany) equipped with a Harrick in situ diffuse reflectance reaction cell to monitor the dynamic evolution of surface intermediates during the catalytic process. Electrochemical impedance spectroscopy (EIS) was performed on a CHI660E electrochemical workstation (Chenhua, Shanghai) to investigate the interfacial charge-transfer behavior.

**XAFS Measurements**

X-ray absorption fine structure (XAFS) spectra at the Ir L_3_-edge were collected at the 44A beamline of the National Synchrotron Radiation Research Center (NSRRC, Taiwan). Measurements were carried out in fluorescence mode using a Lytle detector, while corresponding reference foils were recorded in transmission mode. The sample powders were pressed onto Kapton adhesive tape to form uniform films, whereas reference materials were finely ground and evenly coated on dedicated adhesive supports to ensure consistent thickness.

**XAFS Data Processing and Analysis**

The acquired extended X-ray absorption fine structure (EXAFS) data were analyzed using the Demeter software suite (ATHENA and ARTEMIS modules). Raw spectra were first background-subtracted and normalized to the edge-jump of the absorption step. The resulting χ(k) functions were then Fourier-transformed into R-space with a Hanning window (Δk = 1.0 Å^-1^) to isolate individual coordination shells. Quantitative fitting of the local coordination environment around Ir centers was performed in ARTEMIS using a least-squares refinement approach.

The theoretical scattering paths, including back-scattering amplitudes, phase shifts, and mean free paths, were computed using the FEFF10 code. The EXAFS fitting followed the general expression:


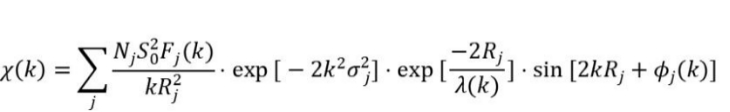


where *S_0_^2^* is the amplitude reduction factor, *N_j_* the coordination number, *R_j_* the average interatomic distance, *F_j_(k)* the effective scattering amplitude, *λ(k)* the mean free path of the photoelectron, *σ_j_* the Debye–Waller factor describing thermal and static disorder, and *ϕ_j_(k)* the total phase shift. This analysis provided quantitative insights into the coordination number, bond length, and structural disorder of Ir centers, revealing the presence of atomically dispersed Ir–N and Ir–S bonds characteristic of the Ir–S/N–C SAzymes.

**Enzyme-like Activity Assays**

For OXD activity, catalytic oxidation of TMB was carried out in the absence of H_2_O_2_. Briefly, 20 μL of Ir–S/N–C SAzymes solution (0.5 mg·mL^-1^) was added into 1960 μL of sodium acetate buffer (0.2 M, pH 3.6), followed by 20 μL of TMB solution (50 mM in DMSO). The mixture was incubated at 30 °C for 15 min, and the absorbance at 652 nm was recorded using a UV–vis spectrophotometer (Shimadzu UV-2600).

For POD activity, the same reaction system was employed with the addition of H_2_O_2_. Typically, 20 μL of Ir–S/N–C SAzymes solution (0.5 mg·mL^-1^) , 20 μL of TMB (50 mM), and 20 μL of H_2_O_2_ ( 50 mM) were mixed with 1940 μL of acetate buffer (0.2 M, pH 3.6). The catalytic oxidation of TMB was monitored at 652 nm. The effects of temperature (20-60 °C) and pH (2-8) on OXD and POD activity were also investigated under identical conditions.

For GSHOx activity, DTNB was used as a probe to detect residual GSH. In a typical assay, 20 μL of Ir–S/N–C SAzymes solution (0.5 mg·mL^-1^) and 15 μL of GSH solution (10 mM) were mixed with in 1950 μL of MES buffer (1.0 M, pH 6.5) at 30 °C for 30 min. Subsequently, 15 μL of DTNB solution (25 mM in DMSO) was added, and the absorbance at 412 nm was measured.

Kinetic analyses of OXD and POD activities were performed by varying the concentration of TMB (0-4 mM) or H_2_O_2_ (0-40 mM) under optimal conditions. Michaelis–Menten curves were obtained, and kinetic parameters (V_max_, K_m_, k_cat._, and k_cat._/K_m_) were calculated by nonlinear fitting using Origin software. The specific activity (U·mg^-1^) of each catalyst was determined following the standard nanozyme activity unit definition, where one unit is defined as the amount of catalyst required to catalyze the oxidation of 1 μmol of TMB per minute under assay conditions.

**Density Functional Theory (DFT) Calculations**

The Gibbs free energy (G) was determined according to the equation:

G = E_DFT_ + E_ZPE_ - TΔS

where E_DFT_ represents the electronic energy derived from DFT calculations, E_ZPE_ denotes the zero-point vibrational energy, and ΔS is the vibrational entropy. All parameters were obtained from first-principles computations. The temperature (T) was fixed at 298.15 K for all simulations. Both the zero-point energy and vibrational entropy were estimated using the harmonic oscillator model, and vibrational frequencies were corrected using standard thermodynamic reference data to minimize basis set-related deviations.

**Colorimetric Detection Assays**

Detection of AA, Cys, and GSH. The OXD-like activity of Ir–S/N–C was used for H_2_O_2_-free detection of reductive molecules. In brief, 20 μL of Ir-S/N-C dispersion (0.5 mg·mL⁻¹) and 20 μL of TMB (50 mM in DMSO) were mixed with 1960 μL of sodium acetate buffer (0.2 M, pH 3.6). After baseline absorbance was recorded at 652 nm (A_0_), 20 μL of different concentrations of AA (0-40 μM), Cys (0-25 μM), or GSH (0-40 μM) were introduced into the reaction system. The absorbance intensity at 652 nm was recorded as A. The relative activity (A-A_0_)/A_0_ ×100% was calculated via linear fitting to the AA, Cys, or GSH concentration. The limit of detection (LOD) was calculated based on the 3σ rule (3σ/slope), and σ is the standard deviation of A for 11 samples.

Detection of AChE activity. Briefly, 200 μL of ATCh solution (8 mM) and 200 μL of AChE solution with varying activity levels (0-10 U·L^-1^) were pre-incubated at 37 °C for 30 min. Then, 20 μL of Ir–S/N–C solution (0.25 mg·mL⁻¹), 20 μL of TMB (25 mM), and 1560 μL of sodium acetate buffer (0.2 M, pH 3.6) were added. After incubation at 37 ℃ for 15 min and the absorbance change at 652 nm was recorded as A. The reaction system in the absence of AChE was recorded as A_0_. The relative activity (A-A_0_)/A_0_ ×100% was calculated via linear fitting to the AChE concentration, and the LOD was determined as described above.

Detection of OPs. Briefly, 200 µL of AChE solution (120 U·L^-1^) and 50 µL of OP standard solution (concentration from 1 ng·mL^-1^ to 1 µg·mL^-1^) were mixed and incubated at 37 °C for 20 min to allow enzyme inhibition. Then, 200 µL of ATCh (8 mM) and 50 µL of Ir–S/N–C SAzymes solution (0.1 mg·mL^-1^) were added to 1490 µL of sodium acetate buffer (0.2 M, pH 3.6). After gentle mixing, 20 µL of TMB solution (25 mM) was introduced, and the reaction mixture was incubated at 30 °C for 15 min. The absorbance change at 652 nm was recorded as A.

Finally, the inhibition rate was calculated as follow equation,

Inhibition (%) = (A - A_0_)/(A_1_ - A_0_) × 100%

where, A_0_ was the absorbance value without OPs, A_1_ was the absorbance value without AChE and OPs. To evaluate selectivity, other pesticides (chlorfenapyr, indoxacarb, tebufenozide, lufenuron, and flufiprole) and common ions (Mg^2+^, Ca^2+^, Zn^2+^, K^+^, Na^+^, SO_4_^2-^, Cl^-^, CO_3_^2-^) were tested under same conditions.

**Detection of OPs in Real Samples**

The practical applicability of the Ir–S/N–C–based colorimetric sensing system was evaluated through the detection of dichlorvos in tap water, apple, and human whole blood, with recoveries determined by the standard addition method. Tap water was collected from the laboratory, filtered through a 0.22 μm fiber membrane, and spiked with OP standard solutions (0.05, 1, and 10 μg·mL^-1^). Fresh apples purchased from a local supermarket were homogenized, and 5 g of the homogenate was mixed with 10 mL acetonitrile, ultrasonically extracted for 20 min, and centrifuged at 5000 rpm for 10 min to collect the supernatant; after spiking with OP solutions (0.05, 1, and 10 μg·mL^-1^), the extract was evaporated to dryness under a stream of nitrogen and redissolved in 1 mL of 0.2 M Tris–HCl buffer (pH 7.5) for testing. Anticoagulated whole blood from healthy volunteers (Chongqing Occupational Disease Prevention and Control Hospital) was pretreated by mixing 100 μL blood with 400 μL acetonitrile, vortexed for 5 min, and centrifuged at 12000 rpm for 15 min; the supernatant was spiked with OPs solutions (0.05, 1, and 10 μg·mL^-1^), evaporated to dryness, and reconstituted in 1 mL Tris–HCl buffer for analysis. For validation, all results were cross-checked using liquid chromatography–mass spectrometry (LC–MS), confirming high accuracy and reliability of the colorimetric method for OP detection in complex matrices.

**Clinical Samples and Ethical Approval**

For clinical validation of the proposed Ir–S/N–C SAzymes-based colorimetric sensing platform, whole blood samples were obtained from healthy adult volunteers at Chongqing Occupational Disease Prevention Hospital, with informed consent obtained from all participants. The study protocol was reviewed and approved by the Ethics Committee of Chongqing Occupational Disease Prevention Hospital (Approval No. 2022–38#). All procedures were conducted in accordance with the ethical standards of the institutional and national research committees and with the 1964 Helsinki declaration and its later amendments. Blood samples were collected using standard anticoagulant tubes and processed immediately for the detection of OPs as described in the analytical protocol.

**Statistical Analysis**

All experiments were performed in triplicate, and results are expressed as mean ± SD. Statistical significance was evaluated by Student’s t-test (for two groups) or one-way ANOVA with Tukey’s post hoc test (for multiple groups). A value of p < 0.05 was considered statistically significant. Kinetic and regression analyses were performed using OriginPro 2022.

**Supplementary Figures**


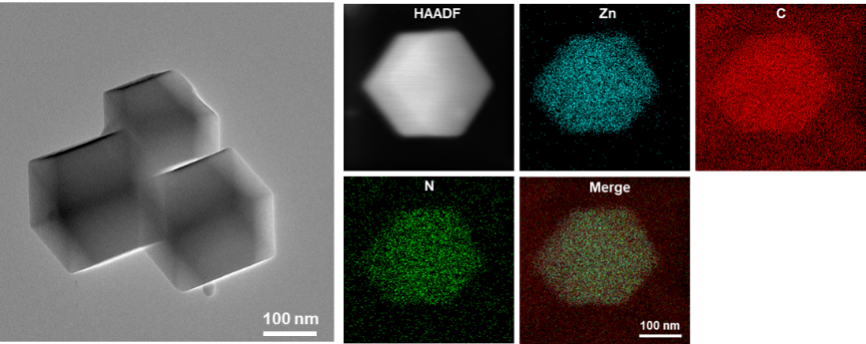


**Figure S1.** TEM images of ZIF-8 and the corresponding EDS mapping images.


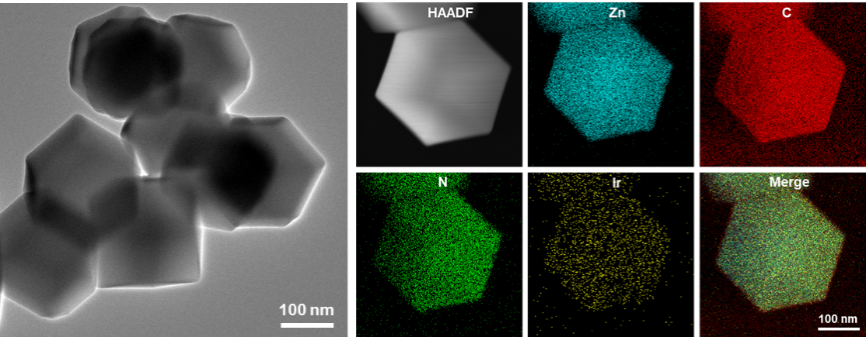


**Figure S2.** TEM images of Ir-ZIF-8 and the corresponding EDS mapping images.


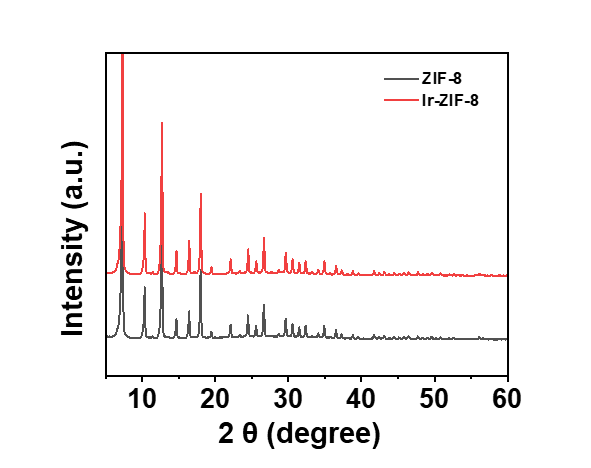


**Figure S3.** XRD pattern of the Ir-ZIF-8 and ZIF-8.


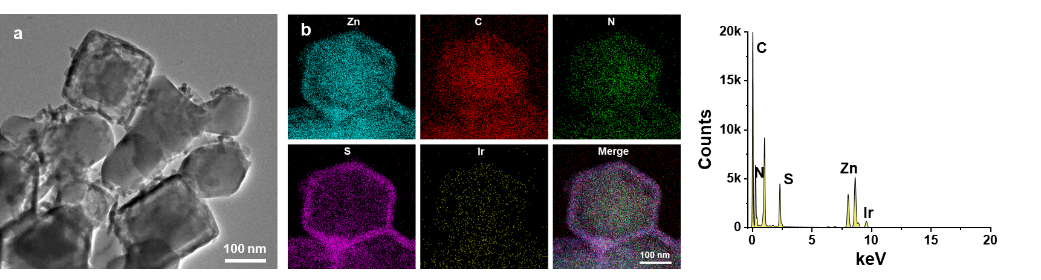


**Figure S4.** (a) TEM images of Ir-ZIF-8@TCA, (b) The EDS mapping of Ir-ZIF-8@TCA and the corresponding EDS spectrum.


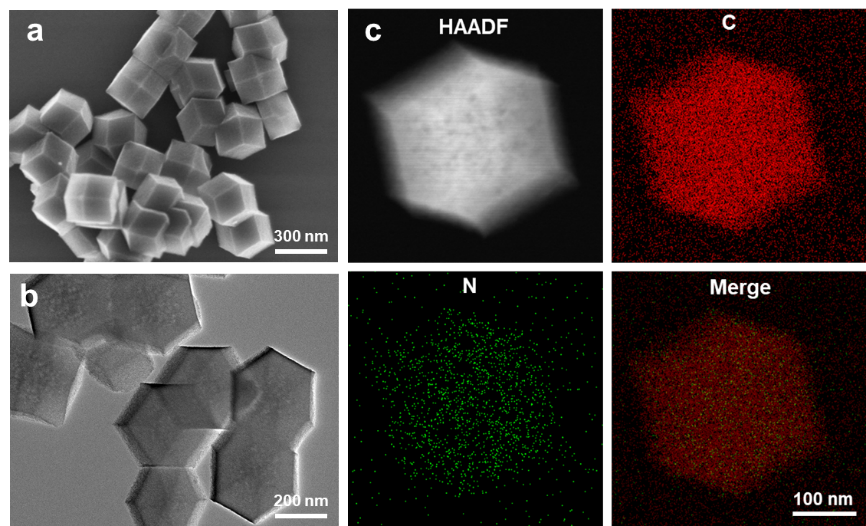


**Figure S5.** SEM (a) and TEM (b) images of N-C control sample, and the corresponding EDS mapping (c).


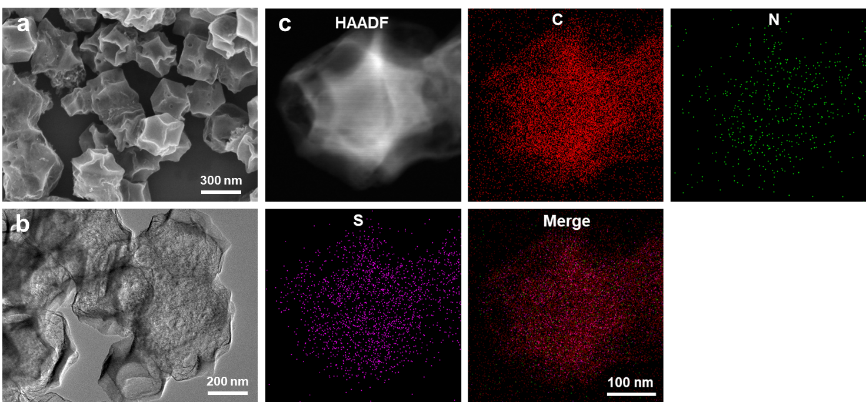


**Figure S6.** SEM (a) and TEM (b) images of S/N-C control sample, and the corresponding EDS mapping (c).


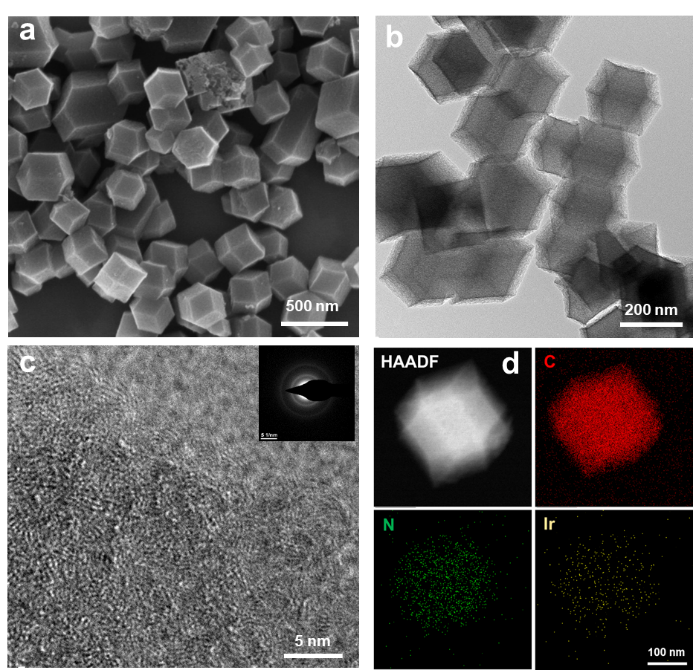


**Figure S7.** SEM (a) and TEM (b) images of Ir-N-C Sazymes. (c) HRTEM image (Inset: SAED pattern) of Ir-N-C SAzyme. (d) Corresponding EDS mapping of Ir-N-C SAzyme.


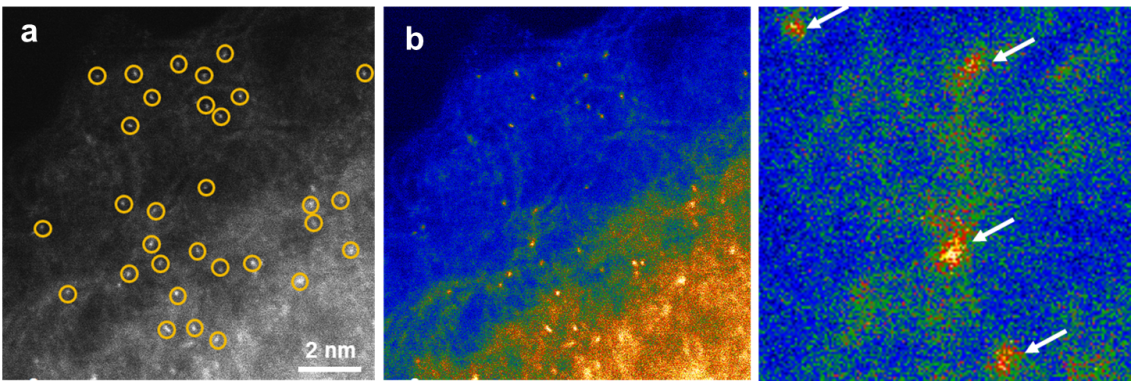


**Figure S8.** (a) Atomic-level HAADF-STEM image of Ir-N-C Sazyme (high-lighted in yellow circles). (b) Corresponding surface intensity map and enlarged surface intensity map of (b), the yellow dots are Ir atoms.


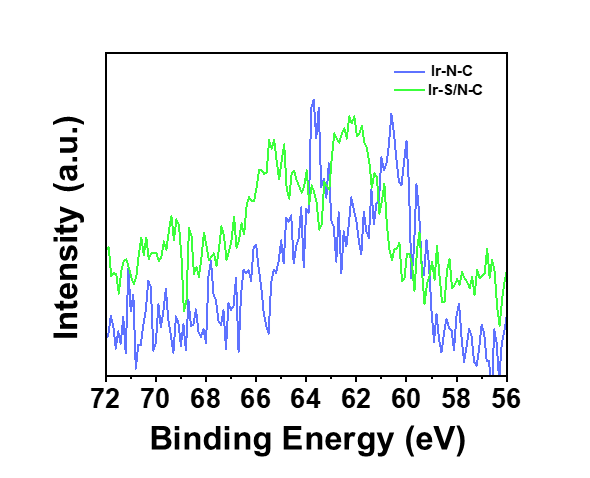


**Figure S9.** High-resolution XPS spectra of Ir 4f orbital of Ir-N-C and Ir-S/N-C SAzymes.


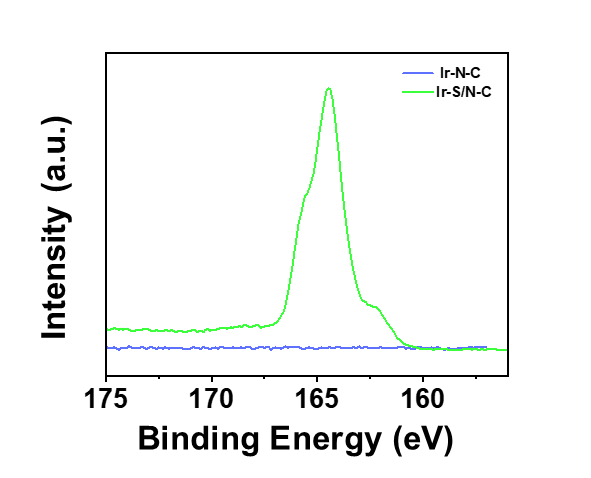


**Figure S10.** High-resolution XPS spectra of S 2p orbital of Ir-N-C and Ir-S/N-C SAzymes.


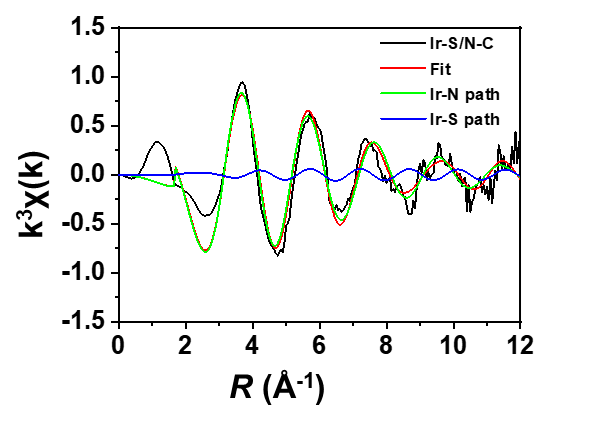


**Figure S11.** The k^3^-weighted EXAFS fitting curves at k space for Ir–S/N–C Sazymes.


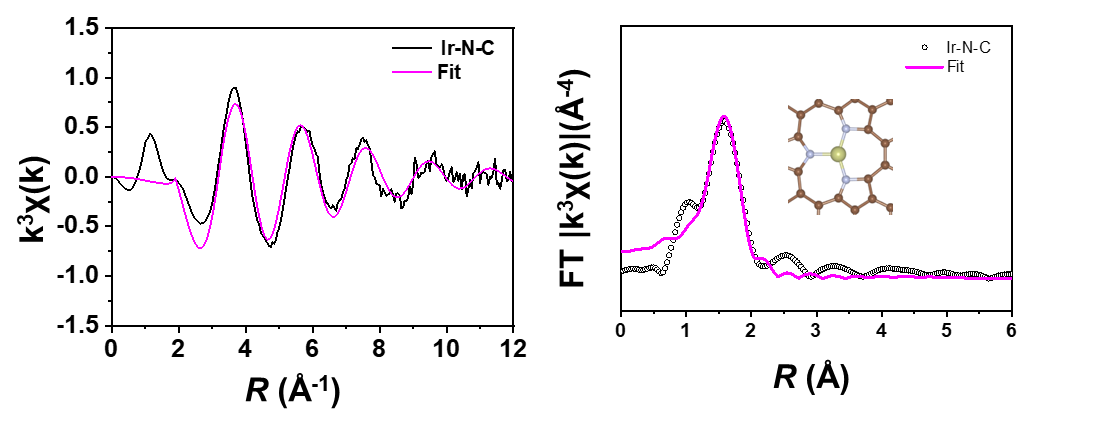


**Figure S12.** Ir L-edge EXAFS analysis of Ir-N-C in K space and R space.


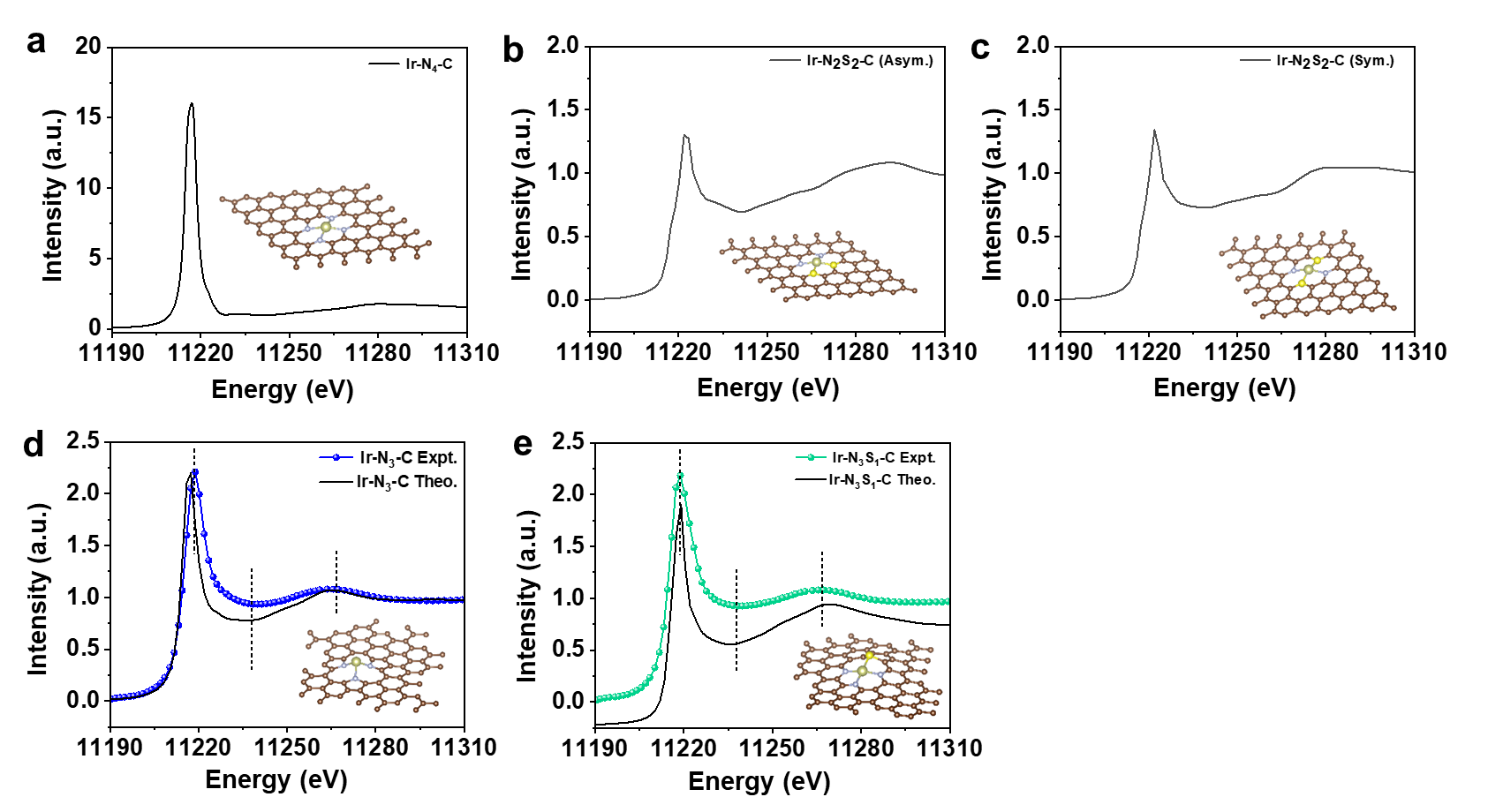


**Figure S13.** Theoretical model comparison and XANES simulation of Ir coordination environments. a–e) DFT-optimized structural models of Ir–N_4_, Ir–N_3_, Ir–N_3_S_1_, Ir–N_2_S_2_ (asymmetric), and Ir–N_2_S_2_ (symmetric), respectively.


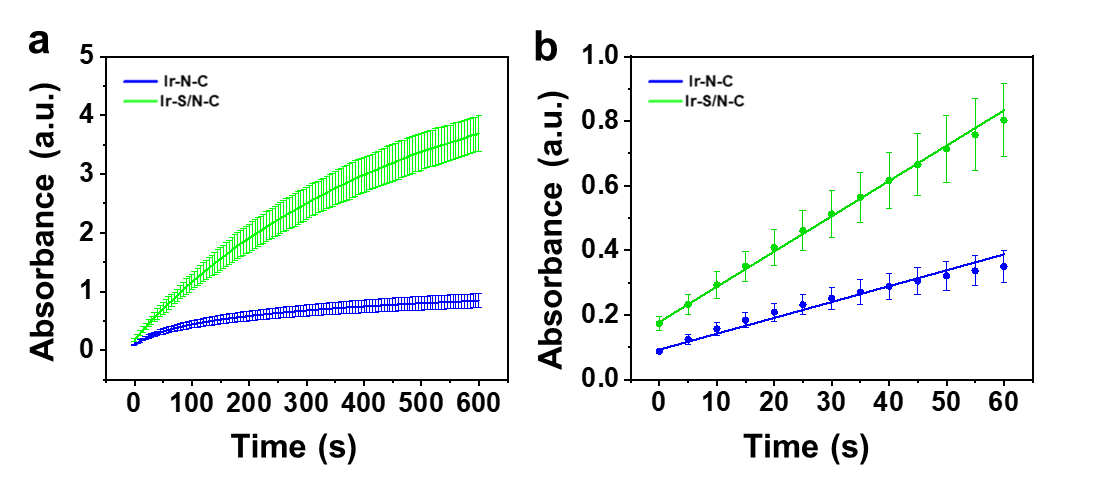


**Figure S14.** a) Reaction-time curves at 652 nm catalyzed by Ir–S/N–C SAzymes with the substrate of TMB. b) The magnified initial linear portion of the reaction-time curves within 60 s.


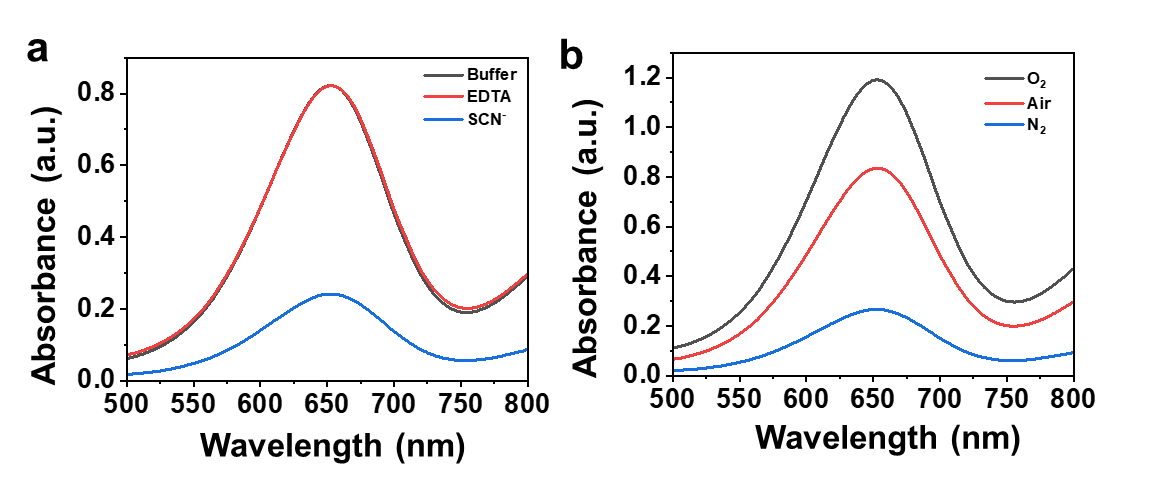


**Figure S15.** a) OXD-like catalytic activity of Ir–S/N–C before and after SCN^-^ poisoning treatment. b) OXD-like catalytic activity of Ir–S/N–C under air, N_2_ and O_2_-saturated conditions.


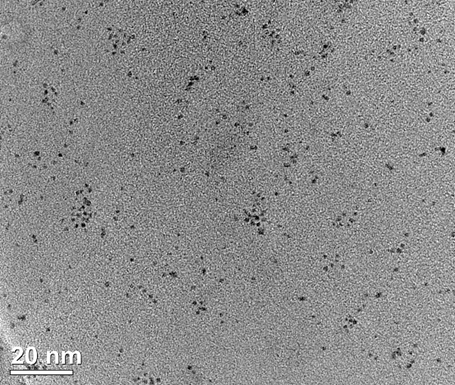


**Figure S16.** TEM images of Ir NPs.


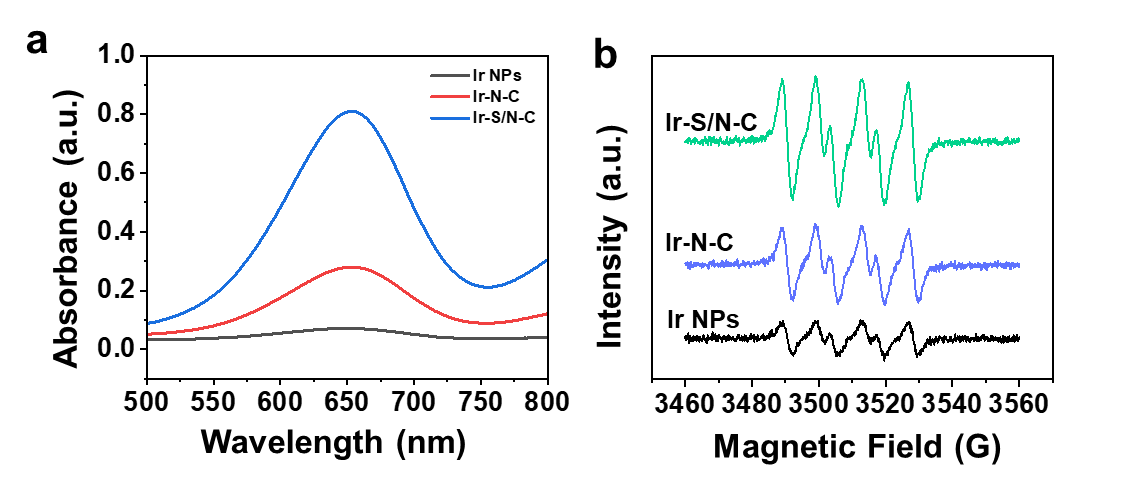


**Figure S17.** a) Comparison of the UV-vis absorption curves toward Ir–S/N–C, Ir–N–C, and Ir NPs, respectively. b) EPR spectra recorded for •O_2_^-^ generation ability of Ir–S/N–C, Ir–N–C, and Ir NPs, respectively.


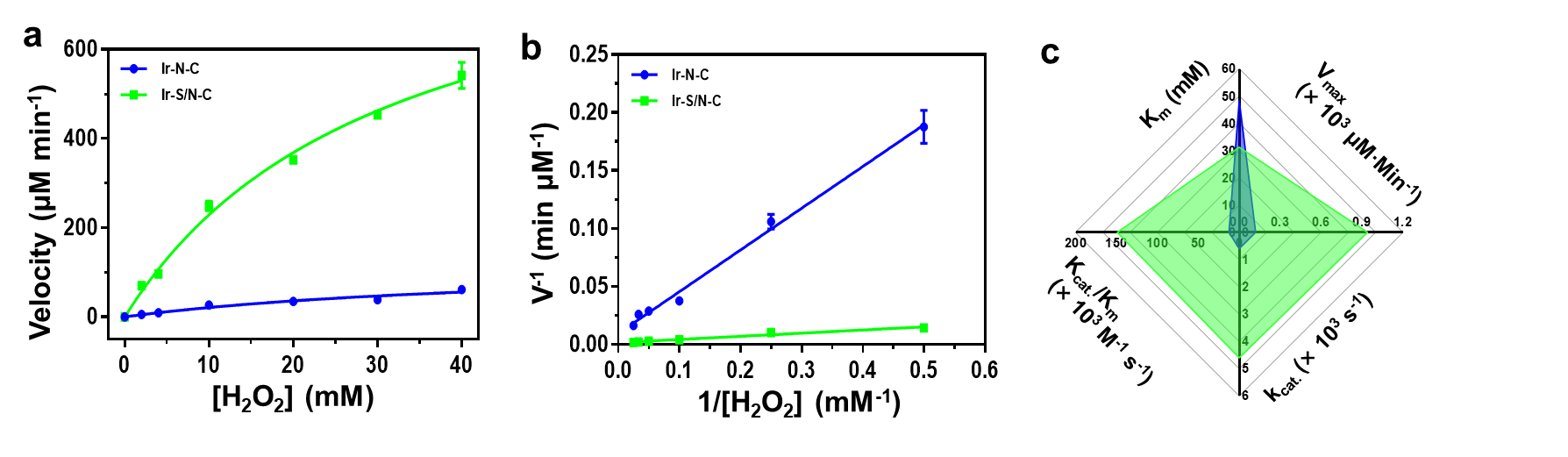


**Figure S18.** a) Michaelis-Menten kinetics of Ir–N–C and Ir–S/N–C SAzymes with H_2_O_2_ substrates, and b) the corresponding Lineweaver-Burk plots. c) Comparison of POD-like kinetics (K_m_, V_max_, k_cat._, and k_cat._/K_m_ values) for Ir–N–C and Ir–S/N–C SAzymes with H_2_O_2_ substrates.


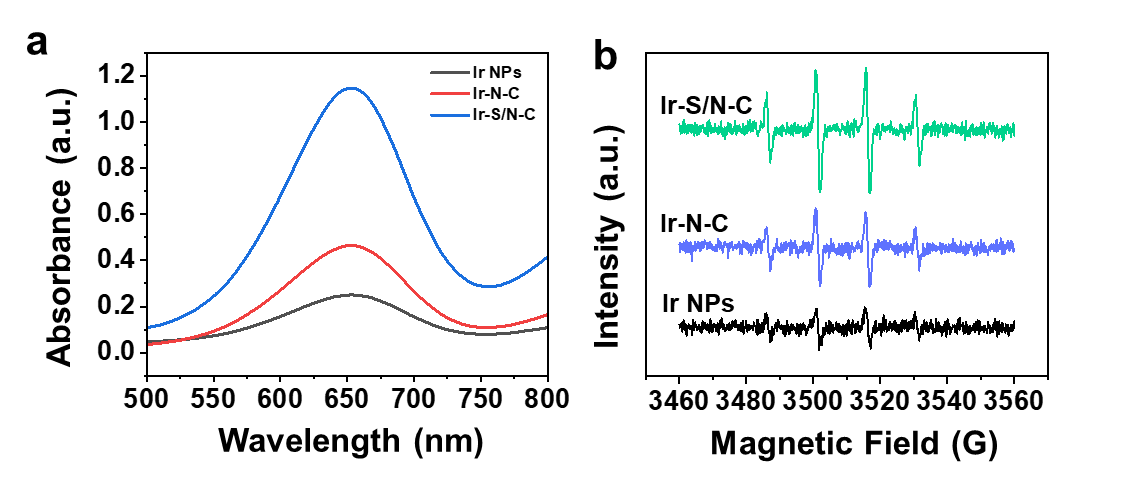


**Figure S19.** a) Comparison of the UV-vis absorption curves toward Ir–S/N–C, Ir–N–C, and Ir NPs, respectively. b) EPR spectra recorded for •OH generation ability of Ir–S/N–C, Ir–N–C, and Ir NPs, respectively.


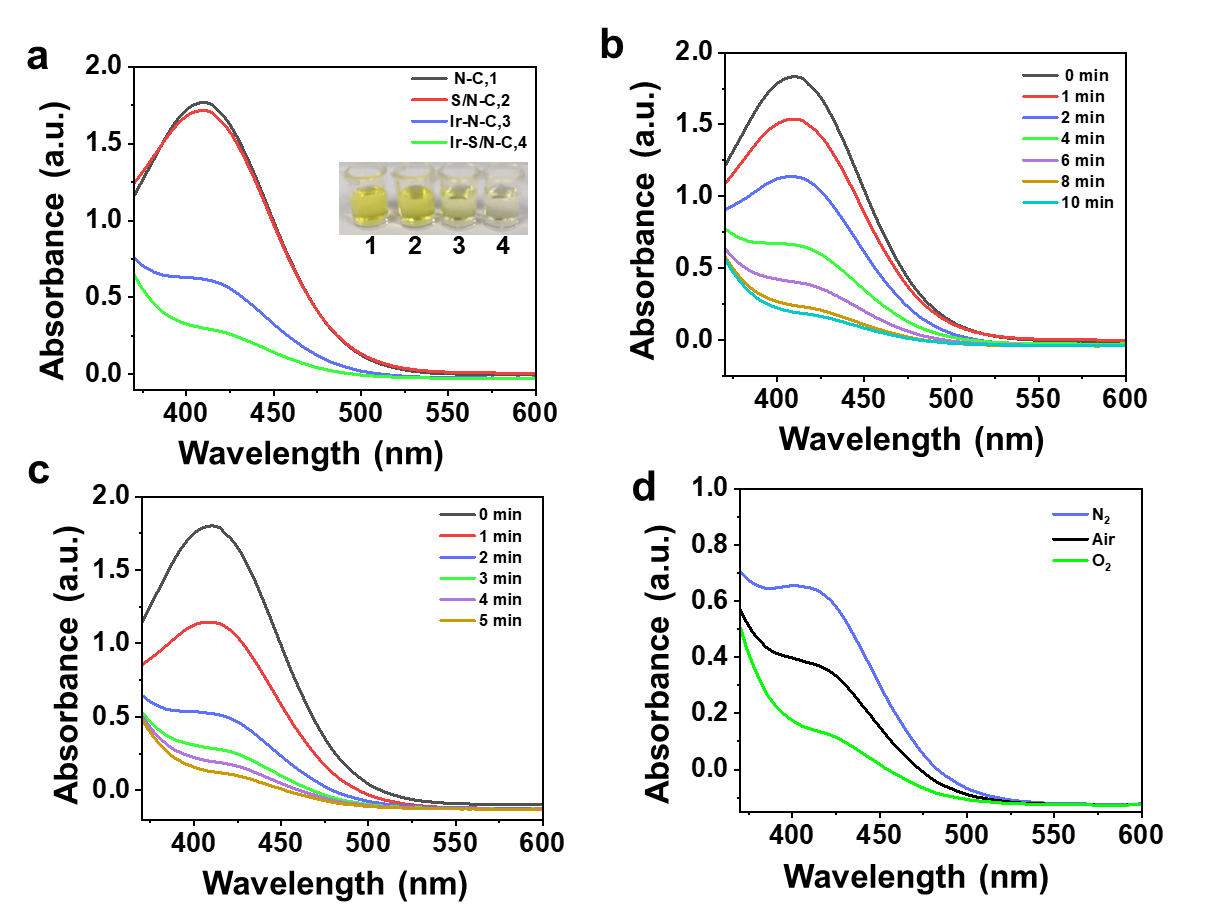


**Figure S20.** (a) UV-vis spectra of GSH consumption after treating with Ir-S/N-C and the comparative samples of N-C, S/N-C, and Ir-N-C, respectively. Time-dependent GSH scavenging by (b) Ir-N-C and (c) Ir-S/N-C detected by UV-Vis spectra. (d) The reduction of GSH catalyzed by the Ir-S/N-C in air, O_2_, and a N_2_ atmosphere, respectively.


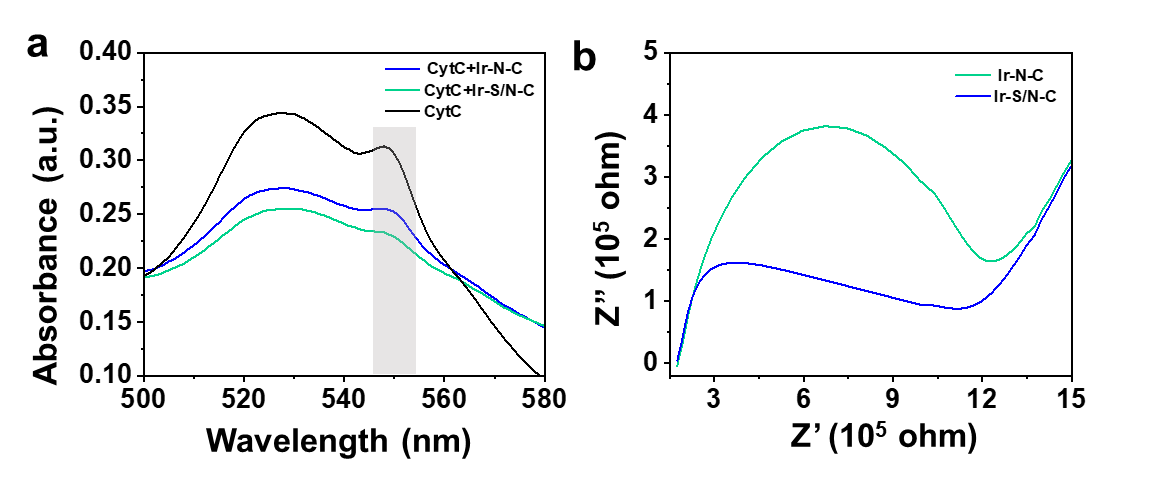


**Figure S21.** (a) UV–vis absorption spectra of Cyt C, Cyt C + Ir-N-C and Cyt C + Ir-S/N-C. (b) EIS curves of Ir-N-C and Ir-S/N-C in 5.0 mM [Fe(CN)_6_] ^3+/4+^ containing 0.1 M KCl.


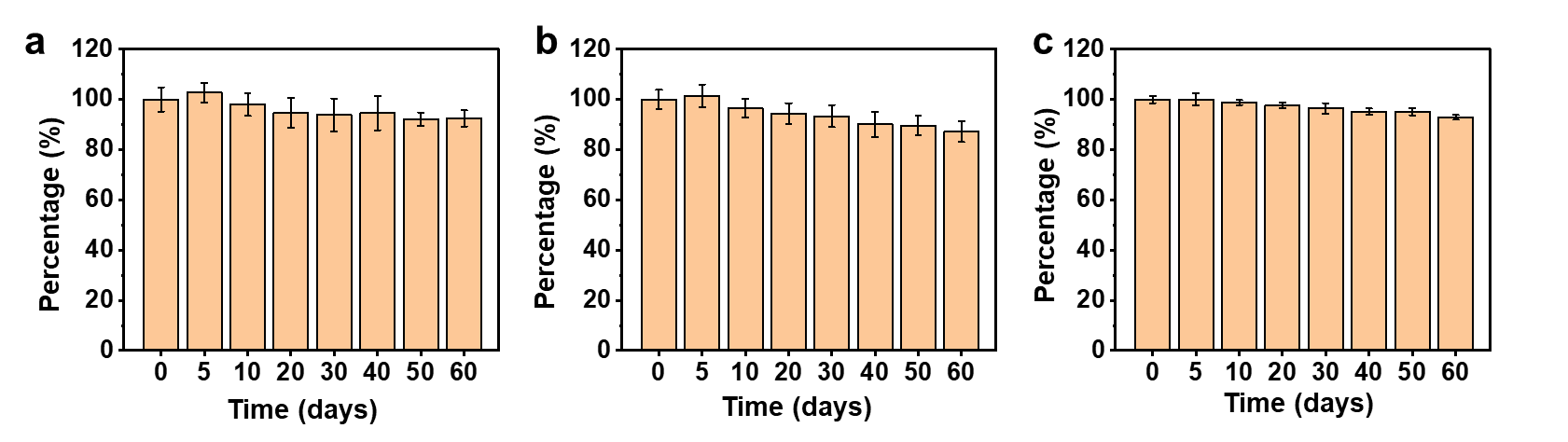


**Figure S22.** Long-term storage stability of the (a) OXD-like, (b) POD-like, and (c) GSHOx-like performance of Ir-S/N-C SAzymes.


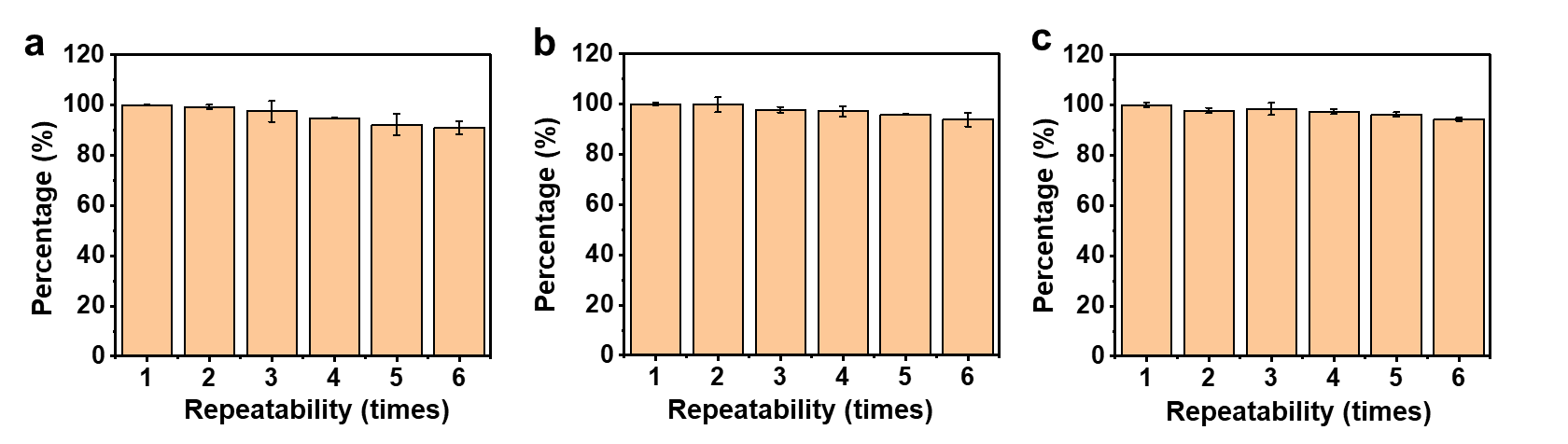


**Figure S23.** Recovery and recyclability of the (a) OXD-like, (b) POD-like, and (c) GSHOx-like performance of Ir-S/N-C SAzymes.


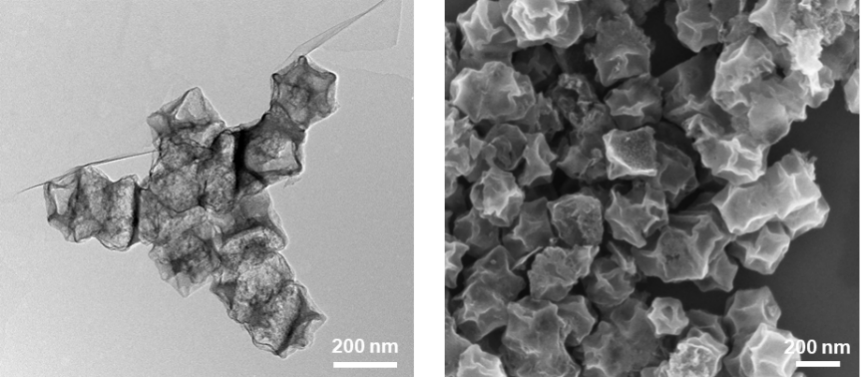


**Figure S24.** The TEM image and SEM image for the Ir-S/N-C SAzyme after multiple enzyme-like reactions.


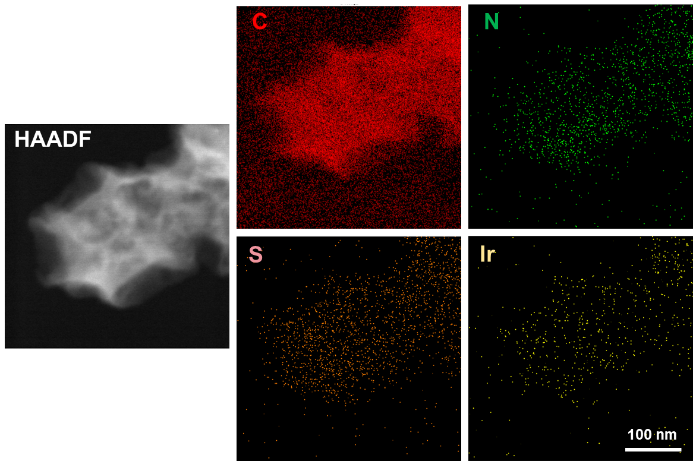


**Figure S25.** The EDX elemental mapping of C, N, S, and Ir elements of the Ir-S/N-C SAzyme after multiple enzyme-like reactions.


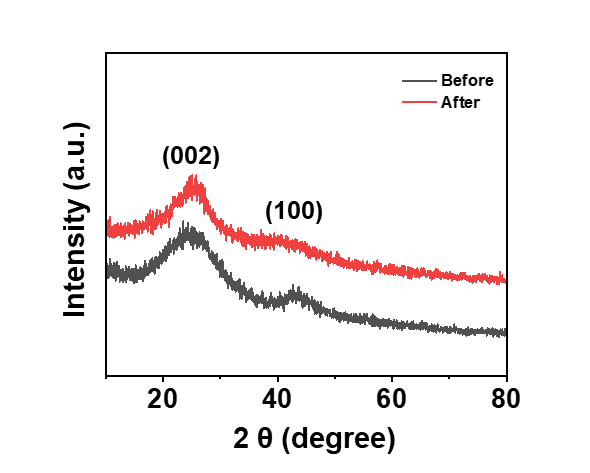


**Figure S26.** The XRD pattern for the Ir-S/N-C SAzyme after multiple enzyme-like reactions.


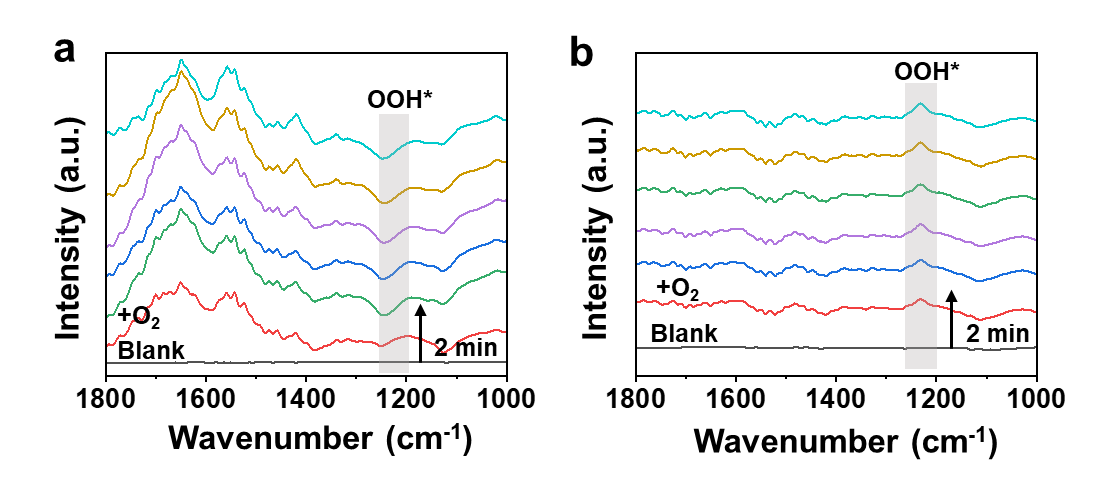


**Figure S27.** In situ ATR-SEIRAS spectra of the catalytic process of O_2_ on (a) Ir-N-C and (b) Ir-S/N-C.


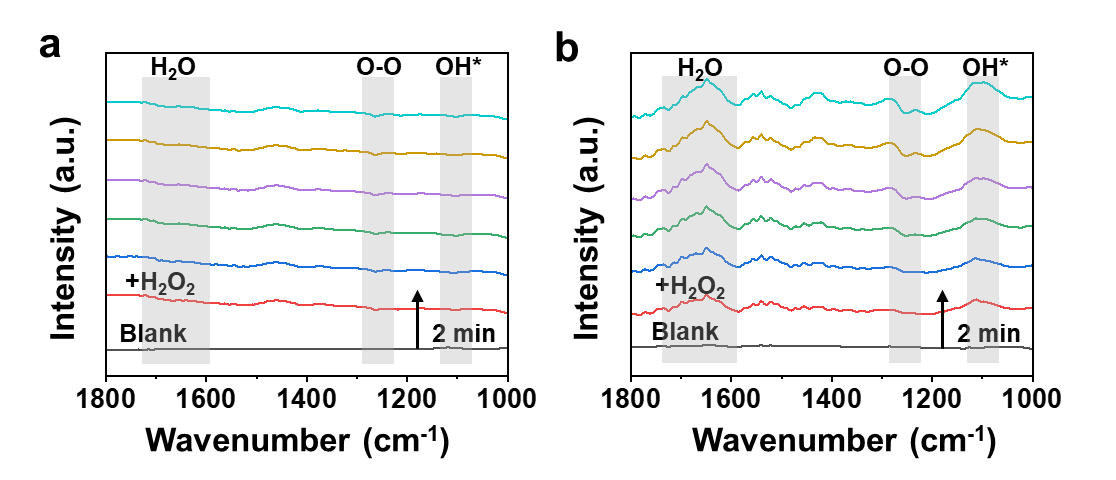


**Figure S28.** In situ ATR-SEIRAS spectra of the catalytic process of H_2_O_2_ on (a) Ir-N-C and (b) Ir-S/N-C.


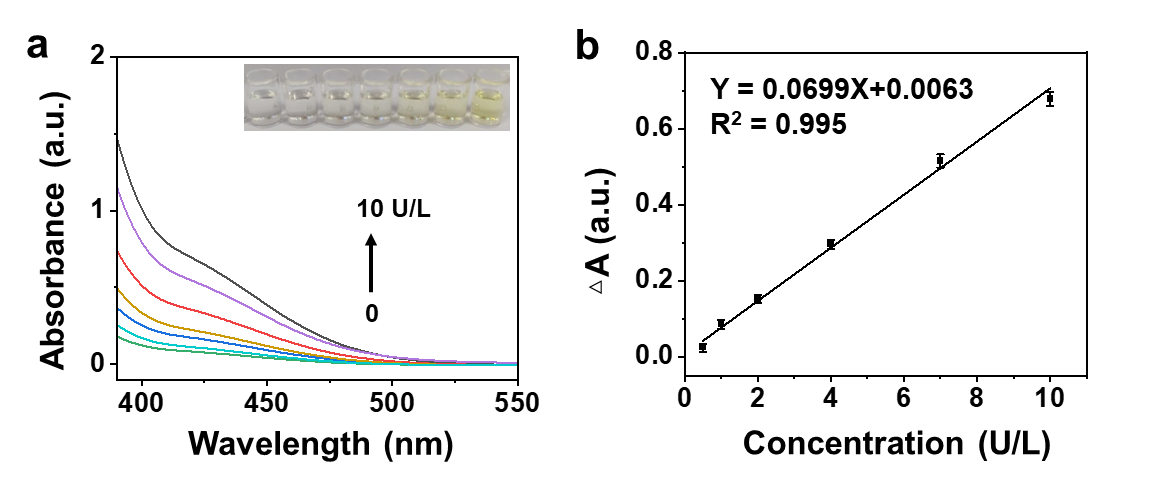


**Figure S29.** (A) UV-vis absorption spectra of DTNB-based assay with various AChE concentrations (B) Plot of absorbance difference at 412 nm (ΔA = A - A_0_, where A and A_0_ represented the absorbance at 412 nm of the sensing system with and without AChE, respectively) versus the AChE concentrations.


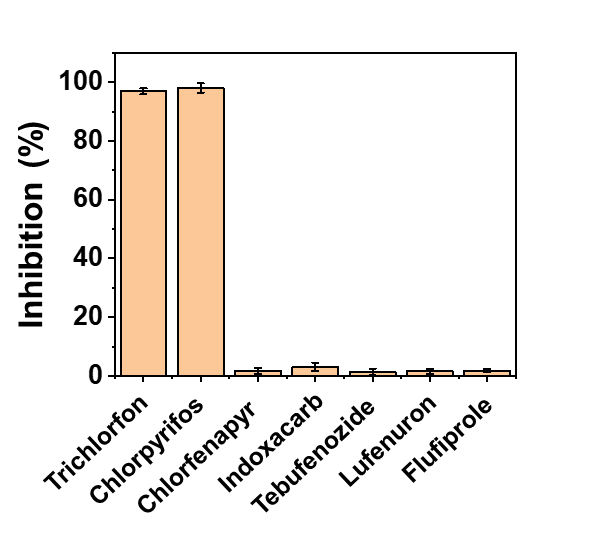


**Figure S30.** Selectivity of the Ir-S/N-C-mediated system for OPs detection.


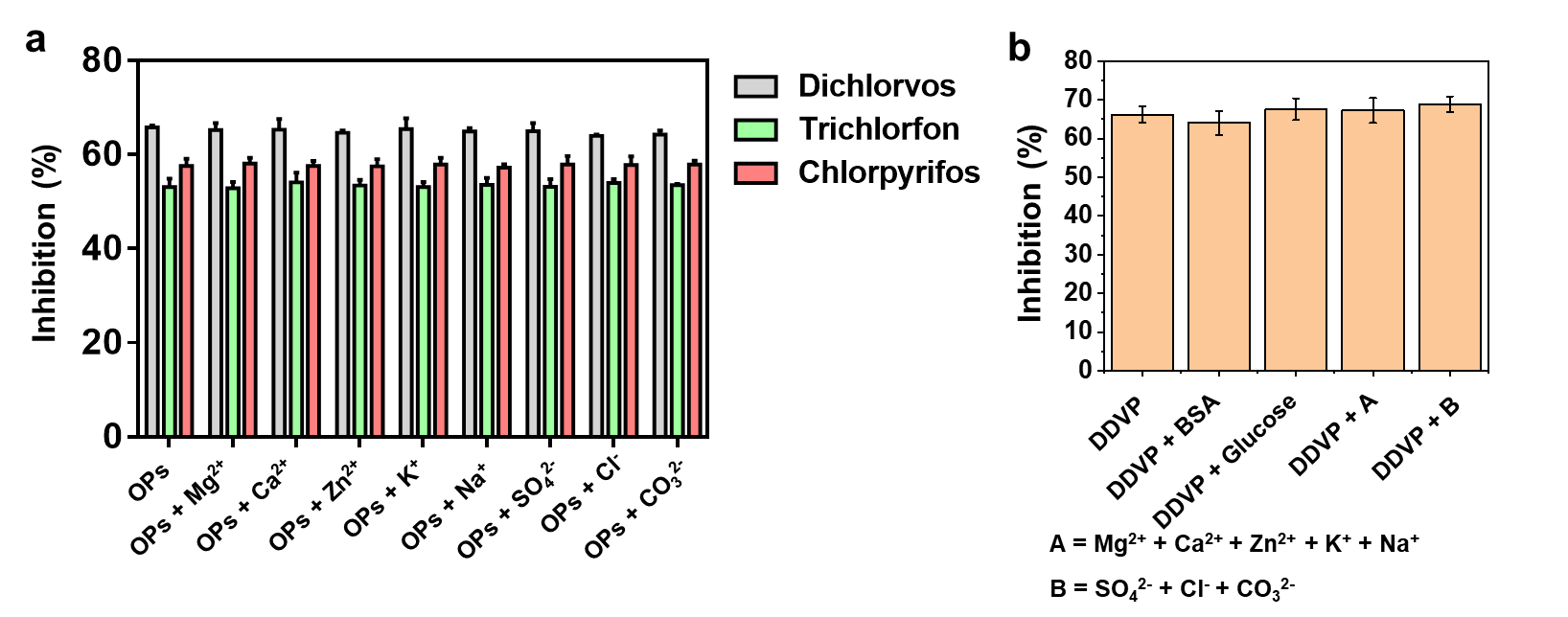


**Figure S31.** a) Anti-interference effect of the Ir-S/N-C-mediated system against various interfering substances. b) Anti-interference effect of the Ir-S/N-C-mediated system in the presence of complex interfering species, including BSA, glucose, mixed cations, and mixed anions.


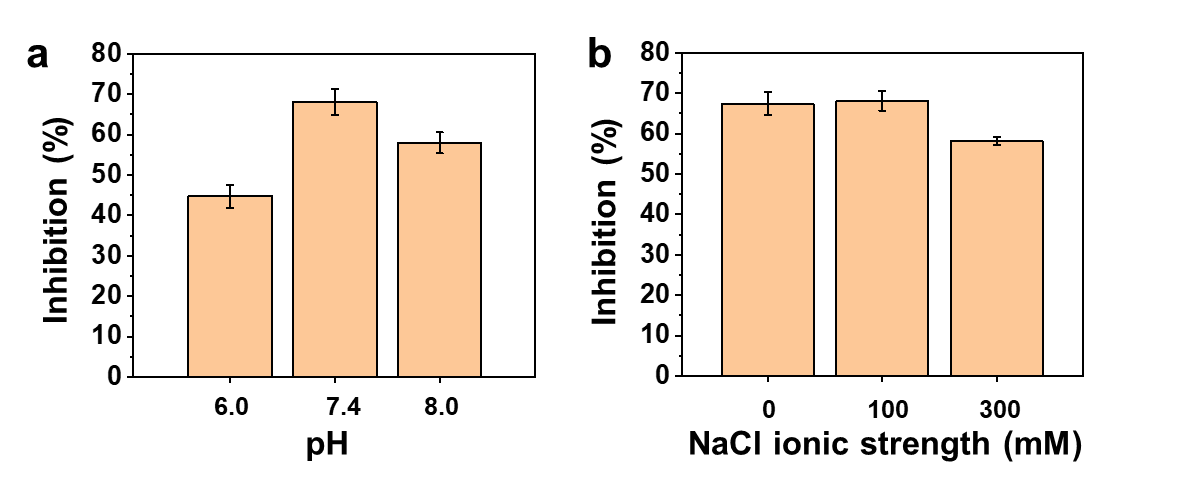


**Figure S32.** Anti-interference performance of the Ir-S/N-C-mediated system in DDV solutions with varying (a) pH and (b) ionic strengths.

**Table S1.** EXAFS fitting parameters at the Ir L-edge various samples (S_0_^2^=0.88)

| samples | path | C. N.^[a]^ | R (Å) ^[b]^ | σ^2^ (×10^-3^ Å^2^) ^[c]^ | ΔE (eV) ^[d]^ | R factor^[e]^ |
| --- | --- | --- | --- | --- | --- | --- |
| Ir-N-C | Ir-N | 3.1 ± 0.4 | 2.01 ± 0.01 | 6.0 ± 2.1 | 6.6 ± 0.7 | 0.011 |
| Ir-S/N-C | Ir-N | 3.1 ± 0.4 | 2.00 ± 0.01 | 6.0 ± 3.4 | 5.3 ± 2.1 | 0.013 |
|  | Ir-S | 0.6 ± 0.2 | 2.51 ± 0.01 | 9.6 ± 2.4 |  |  |

*^a^*C. N.: coordination numbers; *^b^R*: bond distance; *^c^σ*^2^: Debye-Waller factors; *^d^*Δ*E*_0_: the inner potential correction. *^e^R* factor: goodness of fit.

**Table S2.** Apparent activation energies (E_a_) of OXD-like and POD-like catalytic reactions over Ir–N–C and Ir–S/N–C SAzymes, determined from temperature-dependent kinetic measurements using Arrhenius plots.

| **Enzyme-like** | **Samples** | **E_a_ (kJ mol^-1^)** |
| --- | --- | --- |
| OXD | Ir-N-C | 52 |
|  | Ir-S/N-C | 31 |
| POD | Ir-N-C | 42 |
|  | Ir-S/N-C | 24 |

**Table S3**. The detection limit and linear range comparison of Ir-S/N-C for OP detection with some reported colorimetric sensors.

| **Detection sensor** | **Method** | **LOD (ng/mL)** | **Linear range (ng/mL)** | **Ref.** |
| --- | --- | --- | --- | --- |
| MO@FHO | Colorimetric | 0.26 | 0.3-15000 | [1] |
| Co/Mn-MOFs | Colorimetric | 3.4 | 3.5-70.2 | [2] |
| Cu SAs/NC | Colorimetric | 0.79 | 1-450 | [3] |
| BSA-CeO_2_ NCs | Colorimetric | 0.9 | 1-6000 | [4] |
| PANI-MnO_2_ | Colorimetric | 65.9 | 84.5-8450 | [5] |
| FeCo-NC DAzyme | Colorimetric | 4.9 | 16.5-1320 | [6] |
| Mn/Fe-MIL(53) MOF | Colorimetric | 0.74 | 2.6-31.6 | [7] |
| PtCu_3_ alloy NCs | Colorimetric | 0.5 | 1-10000 | [8] |
| MIL-88B(V) | Colorimetric | 20 | 60-10000 | [9] |
| HSA-Au NCs | Colorimetric | 0.44 | 8-1000 | [10] |
| Ir-S/N-C | Colorimetric | 0.85 | 1-1000 | This work |

**References**

[1] M. Zuo, Y. Yang, S. Jiang, et al., “Ultrathin-FeOOH-coated MnO(2) nanozyme with enhanced catalase-like and oxidase-like activities for photoelectrochemical and colorimetric detection of organophosphorus pesticides,” Food Chem 445 (2024): 138716. <https://doi.org/10.1016/j.foodchem.2024.138716>.

[2] J. Liu, X.Y. Chen, J. Chen, “A bimetallic nanozyme with high peroxidase-like activity for visual detection of organophosphorus pesticides,” Talanta 295 (2025): 128309. <https://doi.org/10.1016/j.talanta.2025.128309>.

[3] R. Huang, Z. Dong, W. Zhang, et al., “Cu-N(4) active site sensing platform for point-of-care colorimetric detection of environmental organophosphorus pesticide,” Anal Chim Acta 1382 (2026): 344856. <https://doi.org/10.1016/j.aca.2025.344856>.

[4] Y. Dai, W. Xu, X. Wen, et al., “Smartphone-assisted hydrogel platform based on BSA-CeO(2) nanoclusters for dual-mode determination of acetylcholinesterase and organophosphorus pesticides,” Mikrochim Acta 191 (2024): 185. <https://doi.org/10.1007/s00604-024-06268-6>.

[5] C.L. Yang, L.H. Yu, Y.H. Pang, X.F. Shen, “A colorimetric sensing platform with smartphone for organophosphorus pesticides detection based on PANI-MnO(2) nanozyme,” Anal Chim Acta 1286 (2024): 342045. <https://doi.org/10.1016/j.aca.2023.342045>.

[6] Y. Li, Q. Ma, H. Gong, et al., “Superior oxidase-mimetic activity of FeCo-NC dual-atom nanozyme for smartphone-based visually colorimetric assay of organophosphorus pesticides,” Mikrochim Acta 191 (2024): 368. <https://doi.org/10.1007/s00604-024-06443-9>.

[7] L. Luo, Y. Ou, Y. Yang, et al., “Rational construction of a robust metal-organic framework nanozyme with dual-metal active sites for colorimetric detection of organophosphorus pesticides,” J Hazard Mater 423 (2022): 127253. <https://doi.org/10.1016/j.jhazmat.2021.127253>.

[8] D. Li, J. Li, C. Wu, et al., “Smartphone-assisted colorimetric biosensor for the determination of organophosphorus pesticides on the peel of fruits,” Food Chem 443 (2024): 138459. <https://doi.org/10.1016/j.foodchem.2024.138459>.

[9] N. Du, W. Weng, Y. Xu, et al., “Vanadium-Based Metal-Organic Frameworks with Peroxidase-like Activity as a Colorimetric Sensing Platform for Direct Detection of Organophosphorus Pesticides,” Inorg Chem 63 (2024): 16442-16450. <https://doi.org/10.1021/acs.inorgchem.4c02716>.

[10] K. Yan, C. Wang, Y. Zhang, et al., “Protein-stabilized gold nanoclusters for colorimetric/fluorescence dual-mode detection of organophosphorus pesticides,” Food Chem 498 (2026): 147115. <https://doi.org/10.1016/j.foodchem.2025.147115>.
